# Evolution of *hes* gene family in vertebrates: the *hes5* cluster genes have specifically increased in frogs

**DOI:** 10.1186/s12862-021-01879-6

**Published:** 2021-07-29

**Authors:** Aya Kuretani, Takayoshi Yamamoto, Masanori Taira, Tatsuo Michiue

**Affiliations:** 1 Graduate School of Science, The University of Tokyo, 7-3-1, Hongo, Bunkyo-ku, Tokyo, 113-0033 Japan; 2 Graduate School of Arts and Sciences, The University of Tokyo, 3-8-1, Komaba, Meguro-ku, Tokyo, 153-8902 Japan; 3 Department of Biological Sciences, Faculty of Science and Engineering, Chuo University, 1-13-27 Kasuga, Bunkyo-ku, Tokyo, 112-8551 Japan

**Keywords:** *hes*, *Xenopus*, *Nanorana*, Gene evolution, Gene cluster, Whole genome duplication, Doubly conserved synteny

## Abstract

**Background:**

*hes* genes are chordate homologs of *Drosophila* genes, *hairy* and *enhancer of split*, which encode a basic helix-loop-helix (bHLH) transcriptional repressor with a WRPW motif. Various developmental functions of *hes* genes, including early embryogenesis and neurogenesis, have been elucidated in vertebrates. However, their orthologous relationships remain unclear partly because of less conservation of relatively short amino acid sequences, the fact that the genome was not analyzed as it is today, and species-specific genome duplication. This results in complicated gene names in vertebrates, which are not consistent in orthologs. We previously revealed that *Xenopus* frogs have two clusters of *hes5*, named “the *hes5.1* cluster” and “the *hes5.3* cluster”, but the origin and the conservation have not yet been revealed.

**Results:**

Here, we elucidated the orthologous and paralogous relationships of all *hes* genes of human, mouse, chicken, gecko, zebrafish, medaka, coelacanth, spotted gar, elephant shark and three species of frogs, *Xenopus tropicalis* (*X. tropicalis*), *X. laevis*, *Nanorana parkeri*, by phylogenetic and synteny analyses. Any duplicated *hes5* were not found in mammals, whereas *hes5* clusters in teleost were conserved although not as many genes as the three frog species. In addition, *hes5* cluster-like structure was found in the elephant shark genome, but not found in cyclostomata.

**Conclusion:**

These data suggest that the *hes5* cluster existed in the gnathostome ancestor but became a single gene in mammals. The number of *hes5* cluster genes were specifically large in frogs.

**Supplementary Information:**

The online version contains supplementary material available at 10.1186/s12862-021-01879-6.

## Background

*hes* genes are chordate homologs of *Drosophila hairy* and *enhancer of split* genes, which encode the basic helix-loop-helix (bHLH) transcriptional repressor [1]. These genes are known to have various developmental functions, including Notch signaling target and neurogenesis [2], somitogenesis, and early development of the presumptive midbrain-hindbrain boundary (pre-MHB) [3, 4].

Many *hes*-related genes have been reported in various species. For instance, mammals including human and mouse have seven *hes* genes, and these genes are considered to form gene family [5, 6]. However, the orthologous relationship between species is not still well-known because, for instance, most of *hes*-related genes in zebrafish and medaka are not called as *hes*, but *her*, *hairy-related gene* [7]. It is thought that these intricate naming is caused partly by the large number of the genes and these sequences are relatively small to compare (their total size of the proteins are around 200 aa).

Recently, a number of vertebrate genomic analyses including frogs, *Xenopus laevis* (*X. laevis*) and *X. tropicalis*, have been reported. *Xenopus* includes diploid to dodecaploid species, although polyploidy is considered to be rare in amniotes. *X. tropicalis* has a diploid genome, and *X. laevis* has an allotetraploid genome [8]. The genomic analysis showed that the allotetraploidization was caused by interspecific crosses between two species that have a diploid genome. Thus, *X. laevis* has two subgenomes, called L and S [9, 10].

We previously identified all *hes* genes of *X. tropicalis* and *X. laevis* by phylogenetic analysis and synteny analysis [11]. In brief, for *X. tropicalis*, we revealed the phylogenetic and synteny relationships of all the 18 *hes* genes with human *hes* genes, and renamed them properly. *X. laevis* has 37 *hes* genes: 18 homeologs, one *laevis*-specific gene, *hes5.7*, and a pseudogene, *hes7.4*. Although the number of genes doubled after allotetraploidization, the homeologs of *hes* genes, except for *hes2*, have been conserved in *X. laevis*. In addition, *Xenopus* frogs have more than two paralogs of *hes5*, *hes6*, and *hes7* genes, in contrast to human *hes* genes. In particular, the number of *hes5* genes in *Xenopus* is quite high. Interestingly, they form two clusters, which we call “the *hes5.1* cluster” and “the *hes5.3* cluster”.

Clustered genes such as the Hox gene cluster, human β-globin gene cluster, and human growth hormone (hGH)/chorionic somatomammotropin gene cluster are considered to be formed as a result of gene duplication and divergence [12, 13], and have various notable functions with unique regulatory mechanisms. Similarly, some of *hes* genes are known to be indispensable in neurogenesis [4], and most of *hes* genes are well conserved. In addition, they make two gene clusters at least in *Xenopus*. This implies that the *hes5* cluster also plays an important role during embryogenesis as other cluster genes.

To understand the evolution and role of *hes* genes in vertebrates, especially the clustered *hes* genes, it is important to identify all *hes* genes and reveal the orthologous relationship. In this study, we have elucidated orthologous and paralogous relationships of the *hes* gene family using phylogenetic and synteny analyses of human, mouse, chicken, zebrafish, medaka, three frog species (*X. tropicalis*, *X. laevis* and *Nanorana parkeri*), *Gekko japonicus*, coelacanth, spotted gar, elephant shark, lamprey, and amphioxus. From these analyses, we revealed that *hes* genes are specifically increased in frogs, and also discussed the evolution of the two *hes5* clusters.

## Results

### Classification of *hes* genes in sarcopterygian

**Fig. 1:**
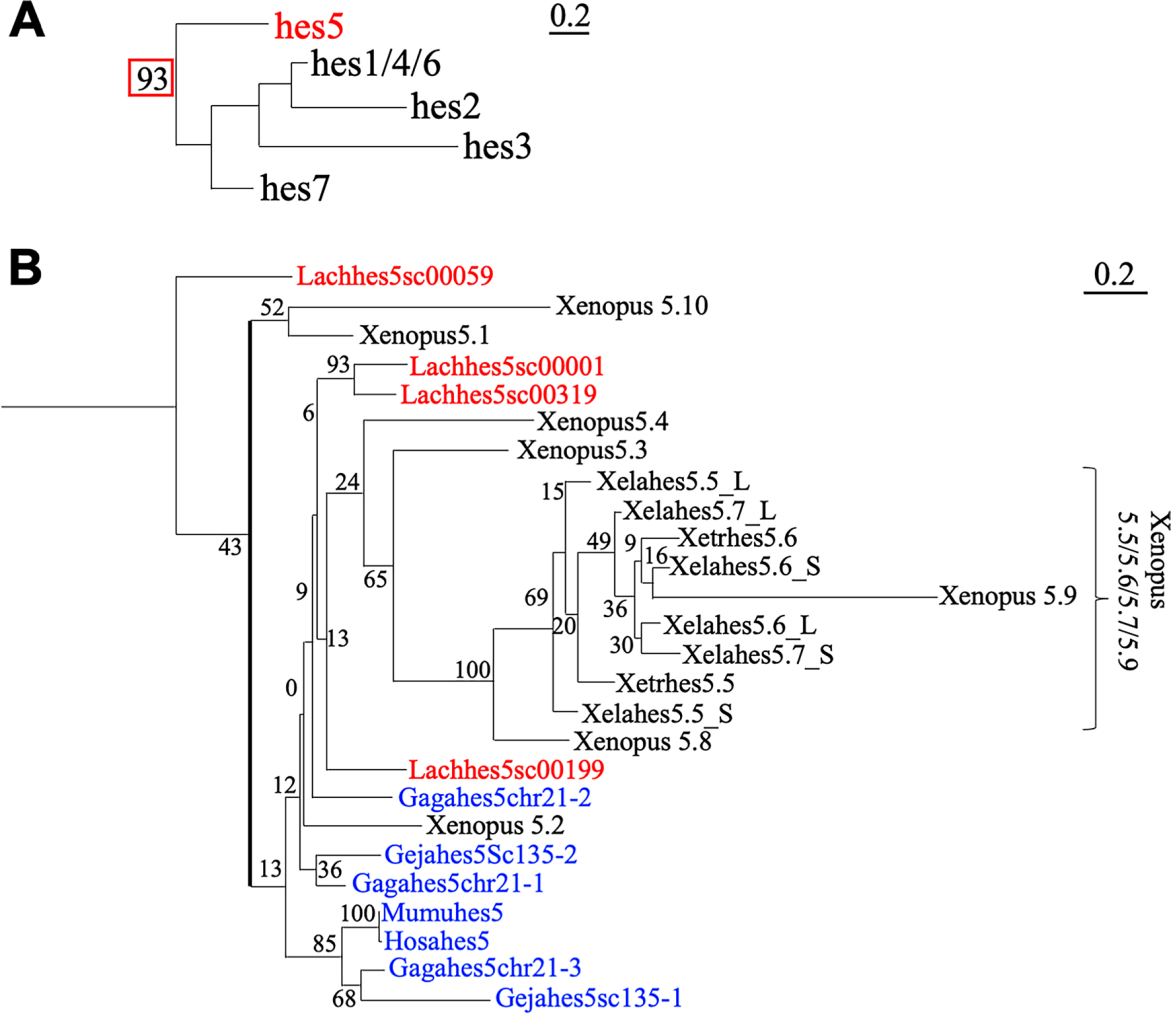
Phylogenetic analysis of *hes* genes in sarcopterygian. **A** The outline of phylogenetic tree in *hes* gene family. **B** The phylogenetic tree was constructed based on the amino acid sequences using the ML method. Bootstrap values for nodes are indicated (n = 100). Evolutionary analysis was conducted in RAxML. Blue and red letters indicate amniote and coelacanth genes, respectively. The abbreviation of animals are as follows: Hosa; human, Mumu; Mouse, Gaga; Chicken, Geja; Japanese gecko, Xetr; *X. tropicalis, Xela; X. laevis*, Lach; Coelacanth

**Fig. 2:**
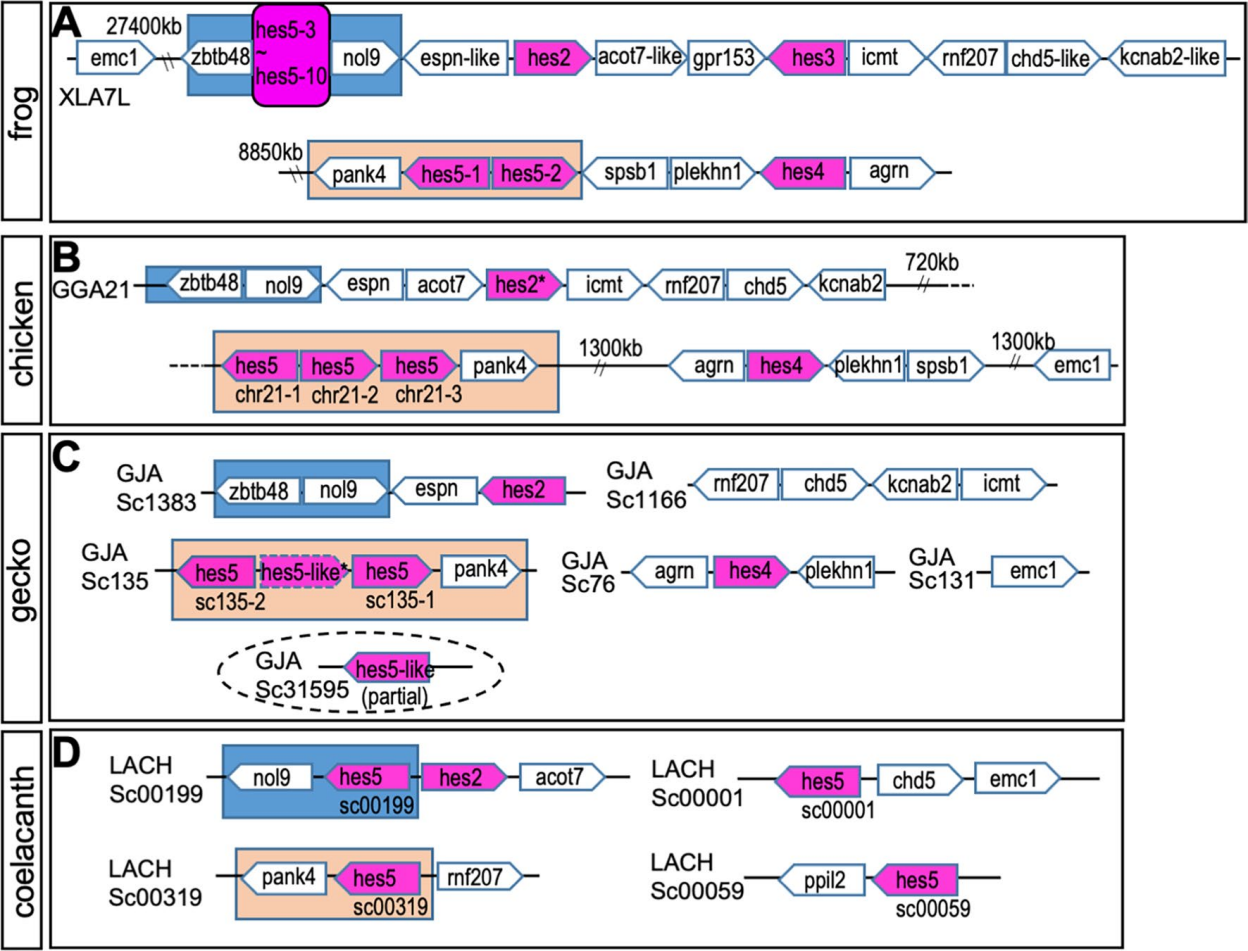
Syntenic analysis of *hes* gene locus in sarcopterygian. The syntenies in frog (**A**), chicken (**B**), gecko (**C**) and coelacanth (**D**) are shown. Chromosome number is described as “XLA7L” in each panel. Pentagon arrows show genes with the 5′–3′ direction. Magenta shows *hes* gene and magenta with a broken line indicates a pseudogene. Orange or blue squares show the *hes5.1* and *hes5.3* cluster region, respectively. The broken lined circle shows “partial” *hes* gene

Our previous studies on the identification of *hes* genes have shown that there are ten *hes5* paralogs in *X. laevis*, which we refer to as "the *hes5.1* cluster" or "the *hes5.3* cluster" [11]. To determine when the *hes5* clusters emerged, we first performed a phylogenetic analysis of sarcopterygian *hes5* genes (Fig. 1, Additional file 1: Fig. S2; complete tree was shown in Additional file 1: Fig. S4). Maximum likelihood (ML) phylogenetic tree construction revealed that all the *hes5* genes we examined were assigned in a single clade with a high bootstrap value (Fig. 1A). In the *hes5* clade, the genes, *Hosahes5*, *Mumuhes5*, *Gagahes5chr21-3* (the name means “Gaga” (*Gallus gallus*) “*hes5*” gene located on “chr21” (chromosome 21), gene number “3”, Fig. 2B), *Gejahes5sc135-1* (*Gejahes5* on scaffold 135, number 1, Fig. 2C), which are human (*Homo sapiens*), mouse (*Mus musculus*), chick (*Gallus gallus*), and gecko (*Gekko japonicus*) genes, formed a single clade with a high bootstrap value. Interestingly, *Xenopus hes5.3-5.9* formed a monophyletic group, suggesting that gene duplication of *hes5.3-5.9* occurred independently (Fig. 1B, blue letters). In Coelacanth (*Latimeria chalumnae* (*Lach*)), one of *hes5* genes formed a single clade (*Lachhes5sc00059*), three genes (*Lachhes5sc00001*, *Lachhes5sc00319* and *Lachhes5sc00199*) formed a clade with *Xenopus hes5.3-5.9* although the bootstrap value was too low. This result suggests that three coelacanth genes may be related to the *hes5.3* cluster.

Next, to examine the presence of the *hes5* clusters in other species, we analyzed synteny of *hes5* locus in chicken, geckos, and coelacanth genomes (synteny of other *hes* genes are shown in Additional file 1: Fig. S1A, C). In the chicken genome, *hes5* genes were located on a single chromosome, chromosome 21 (Fig. 2B). In gecko, synteny around *hes5* was observed in scaffolds 135 and 31595 (Fig. 2C). In coelacanth, we found four *hes5* genes in scaffold00199, 00001, 00319, and 00059 (Fig. 2D). *Hes5chr21-1*~*3* genes in chick, *hes5sc135-1*~*2*, and *hes5-like* genes in gecko, and *hes5sc00319* gene in coelacanth were all located next to *pank4*, similar to *Xenopus hes5.1* cluster genes. This suggests that these genes correspond to the *hes5.1* cluster (orange background). In chicken and gecko, however, there were no *hes5* genes between *nol9* and *zbtb48* (the *hes5* genes located between these genes are defined as *hes5.3* cluster genes in *Xenopus*, blue background). In contrast, the coelacanth *Lachhes5sc00199* gene was located near *nol9*. This result suggested that coelacanth *Lachhes5sc00199* may be homologous to the *hes5.3* cluster gene. In coelacanth, *Lachhes5sc00001* was found near *chd5* (Fig. 2D). In *Xenopus*, *chd5* (*chd5-like*) was located next to *rnf207* near the *hes5* clusters, suggesting the relevance of the coelacanth gene to the *hes5* clusters. *Lachhes5c00059* was found near *ppil2*, which is located on the 1st chromosome in *Xenopus*, indicating that the synteny was different from other *hes5* genes. Phylogenetic analysis also indicated that *Lachhes5sc00059* was first divided in the *hes5* gene family (Fig. 1B), suggesting a distinct evolution of this gene. Together with these results, it is suggested that all the *hes5* genes of chicken and gecko are classified to the *hes5.1* cluster*,* whereas coelacanth *hes5* genes belong to the *hes5.1* and *hes5.3* clusters.

### Comparison of *hes* genes between teleosts and *Xenopus*

**Fig. 3:**
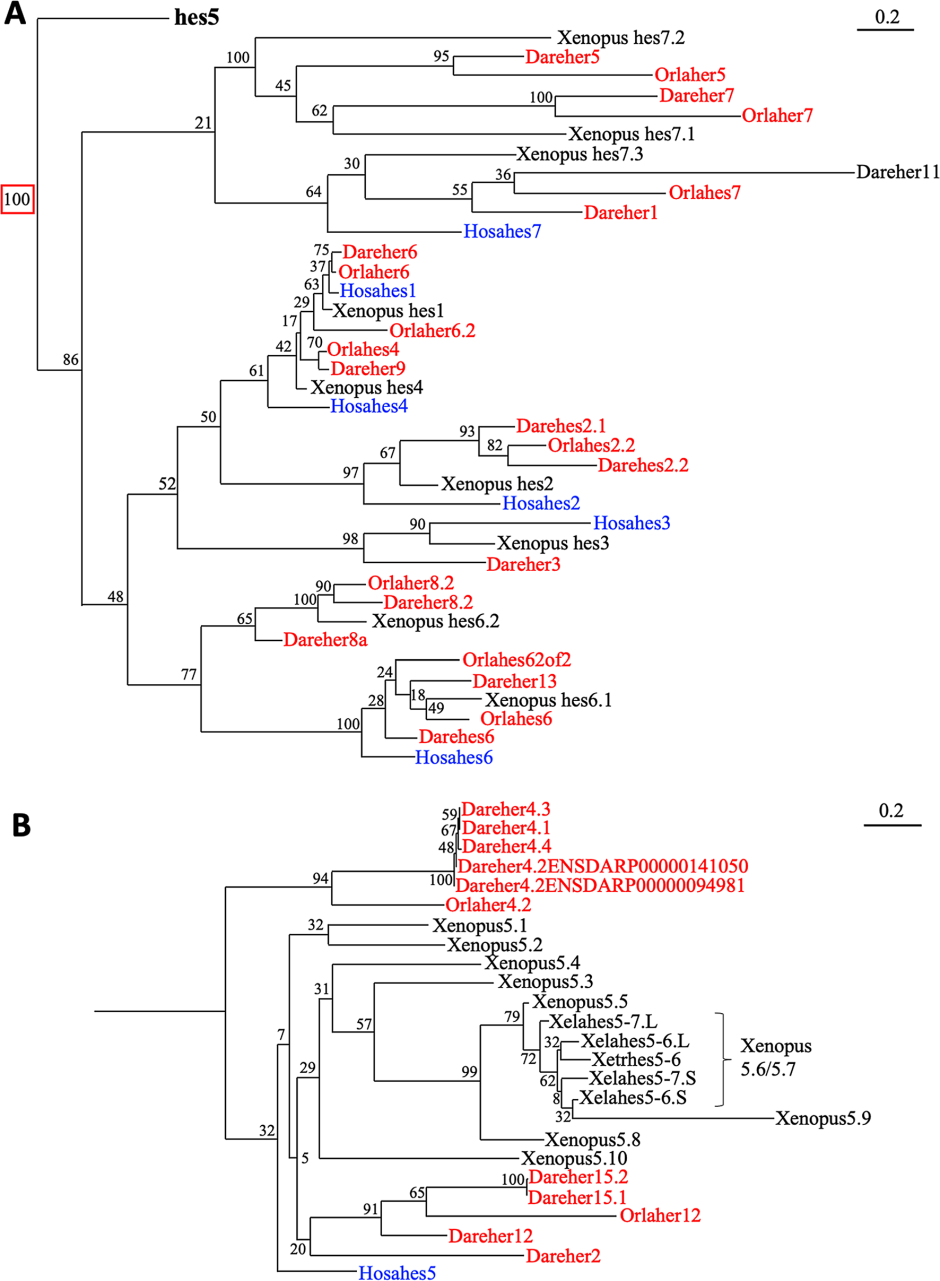
Phylogenetic analysis of teleost *hes* genes. The phylogenetic tree was constructed by ML method. *hes* genes except for *hes5* (**A**), only *hes5* genes (**B**). Red and blue letters indicate zebrafish/medaka and human *hes* genes, respectively. Hosa, human; Xela, *Xenopus* laevis; Xetr, *Xenopus tropicalis*; Dare, zebrafish; Orla, medaka

It is known that whole genome duplication (WGD) occurred 500 million year ago in the common ancestor of vertebrates. Additionally, in teleost, another WGD occurred 3.7 million years ago after divergence from the common ancestor of gnathostomes [14, 15]. Thus, in teleost genome, the two loci having similar gene order to each other on different chromosomes are called doubly conserved synteny (DCS), are found and the fact that the gene is doubly conserved even after WGD suggests that it has an important function [16]. In zebrafish (*Danio rerio*) and medaka (Japanese ricefish, *Oryzias latipes*), *hes* genes have not been well characterized, especially the orthologous relationship between species. Indeed, many genes that seem to be *hes* orthologues were named as "*her*" genes. Therefore, we attempted to identify the orthologous relationship of teleost *hes* genes based on phylogenetic analysis by their amino acid sequences. By our phylogenetic analysis, we found that many zebrafish and medaka “*her*” genes formed clades with *Xenopus hes* genes (Fig. 3A; complete tree is shown in Additional file 1: Fig. S5; detailed gene annotations are shown in Additional file 1: Table S1). Zebrafish *her6* and medaka *her6*, *her6.2* formed a single clade with *Xenopus hes1*. Medaka *her4* and zebrafish *her9* were located near *Xenopus hes4* clade. *Orlaher8.2* and *Dareher8.2, 8a* showed high similarity with *Xenopus hes6.2*. *Dareher13*, *Darehes6*, *Orlahes62of2*, and *orlahes6* belonged to a clade of *Xenopus hes6.1* and human *hes6* genes. The genes annotated as *hes3* and *hes2* in zebrafish, medaka, and *Xenopus* belonged to each homologous clade. *her5, 7* of both medaka and zebrafish belonged to *Xenopus hes7.1* clade, whereas zebrafish *her1*, *her11*, and medaka *her7* belonged to *Xenopus hes7.3* clade.

About *hes5* genes, many homologous genes were found in both zebrafish and medaka, *Dareher4.1-her4.4*, two genes named *Dareher4.2*, *Dareher2, 12, 15.1-15.2*, and *Orlaher4.2, 12* (Fig. 3B). These genes formed teleost-specific monophyly among the large *hes5* clade. Thus, it is not clear which *Xenopus hes5* cluster zebrafish/medaka *hes5* belongs to.

**Fig. 4:**
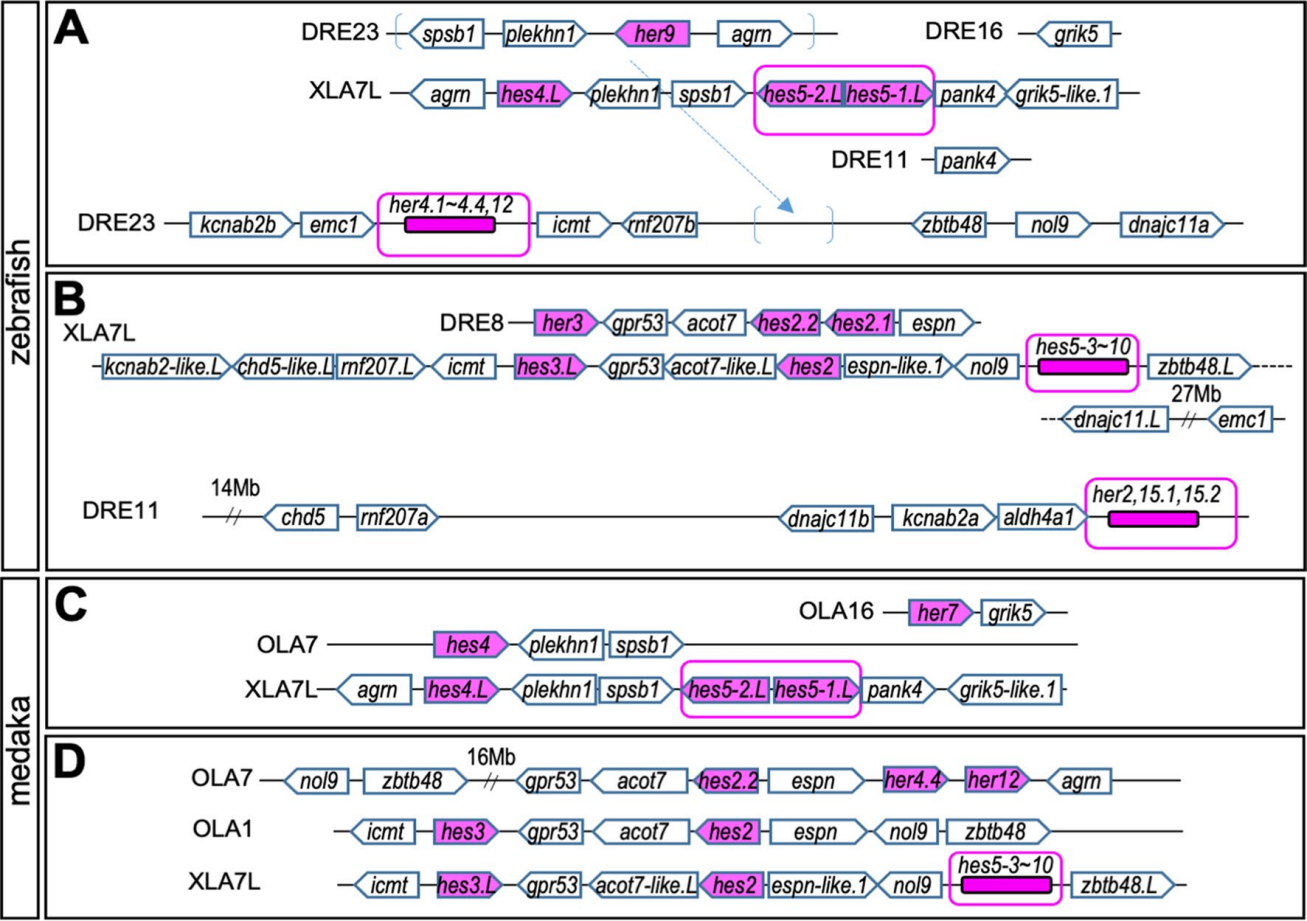
Comparison with *hes* gene locus among *Xenopus*, zebrafish and medaka. Chromosome number is described as “DRE23”. Pentagon arrows show genes a gene with 5′–3′ direction. Magenta shows *hes* gene. Broken arrow means same region (on DRE23). DRE, zebrafish; OLA, medaka; XLA, *Xenopus laevis*

We next performed synteny analysis around *her4.1*-*4.4, 12* and *her2, 15.1-15.2* to clarify whether these genes formed clusters similar to *Xenopus hes5.1* and *hes5.3* clusters (the synteny of other teleost *hes* genes were shown in Additional file 1: Fig. S1B, D). In the zebrafish genome, *her4.1*-*4.4, 12* cluster and *her2, 15.1-15.2* cluster were present on chromosomes 23 and 11, respectively. *dnajc11* and *rnf207* genes were found in the genomic region around the clusters. *Icmt*, *kcnab2*, *nol9*, and *chd5* genes located in *Xenopus hes5* locus were also found on either chromosome 23 (DRE23) or chromosome 11 (DRE11). These results suggested that DCSs were found in the *hes5* region of the zebrafish genome. Near the *her2, 15* cluster on DRE11, *dnajc11*, which is located near the *hes5.3* cluster in *Xenopus*, was found (Fig. 4A). However, other typical features of the *hes5.3* cluster were not observed in the locus. For instance, *nol9* or *zbtb48* was not located near the *hes2, 15* locus. On DRE23, the *her4.1-4.4, 12* cluster was located between *emc1* and *icmt* (Fig. 4A). *Icmt* gene was located near *hes3* (*hes3.L*) gene in *Xenopus* (Fig. 4B). No *her* or *hes* gene was found between the locus of *zbtb48* and *nol9*, as in the chicken (Fig. 2B). From these results, it was difficult to determine whether the *her4.1-4.4, 12* cluster corresponds to the *hes5.1* cluster or *hes5.3* cluster in *Xenopus*. It should be pointed out that the sequence homology of the zebrafish genes with *Xenopus hes5* genes appeared to be higher with the *hes5.1* cluster genes than with the *hes5.3* cluster genes (Table 1), suggesting that *her2, 15* and *her 4.1-4.4, 12* genes of zebrafish might correspond to the *hes5.1* cluster genes in *Xenopus*. In medaka, *her7* gene was found to be located near *grik5*, which was located near the *hes5.1* cluster in *Xenopus* (Fig. 4C). However, phylogenetic analysis showed that *OLA her7* was in *Xenopus hes7.1* subclade (Fig. 3A). On the other hand, *OLA her4.4* and *her12* were located on medaka chromosome 7 around the following genes, *espn*, *acot7*, and *hes2.2*, which were located near the *hes5.3* cluster in *Xenopus*. No *hes*-related genes were located between *nol9* and *zbtb48* (Fig. 4D, medaka chromosome 1) although similar gene order to *Xenopus hes5* region was also observed around the locus (Fig. 4D).

**Table 1:** *hes5* protein sequence identity between zebrafish and *Xenopus*

	*hes5.1*	*hes5.2*	*hes5.3*	*hes5.4*	*hes5.5*	*hes5.6*	*hes5.8*	*hes5.9*	*hes5.10*
*Dareher4.4*	41.96%	40.82%	37.68%	37.41%	35.46%	34.33%	36.88%	28.89%	31.25%
*Dareher4.3*	41.96%	40.82%	38.41%	37.41%	35.46%	34.33%	36.88%	28.89%	31.25%
*Dareher4.2* (*ENSDARP00000094981*)	41.96%	40.82%	37.68%	37.41%	32.91%	31.79%	36.88%	28.89%	31.25%
*Dareher4.2* (*ENSDARP00000141050*)	41.96%	40.82%	37.68%	37.41%	32.91%	31.79%	36.88%	28.89%	31.25%
*Dareher4.1*	41.96%	40.82%	37.68%	37.41%	35.46%	34.33%	36.88%	28.89%	31.25%
*Dareher2*	51.46%	53.27%	48.49%	54.55%	39.17%	43.48%	47.57%	33.88%	45.61%
*Dareher15.2*	47.18%	44.60%	39.58%	41.43%	36.84%	38.52%	40.74%	38.51%	37.58%
*Dareher15.1*	45.51%	42.04%	39.58%	42.14%	36.84%	35.76%	40.00%	37.84%	37.58%
*Dareher12*	47.22%	44.37%	41.89%	41.73%	40.76%	37.82%	45.52%	36.71%	40.12%

Zebrafish gene names in the vertical direction; *Xenopus* gene names in the horizontal direction

### Classification of *hes* genes in gnathostomata

**Fig. 5:**
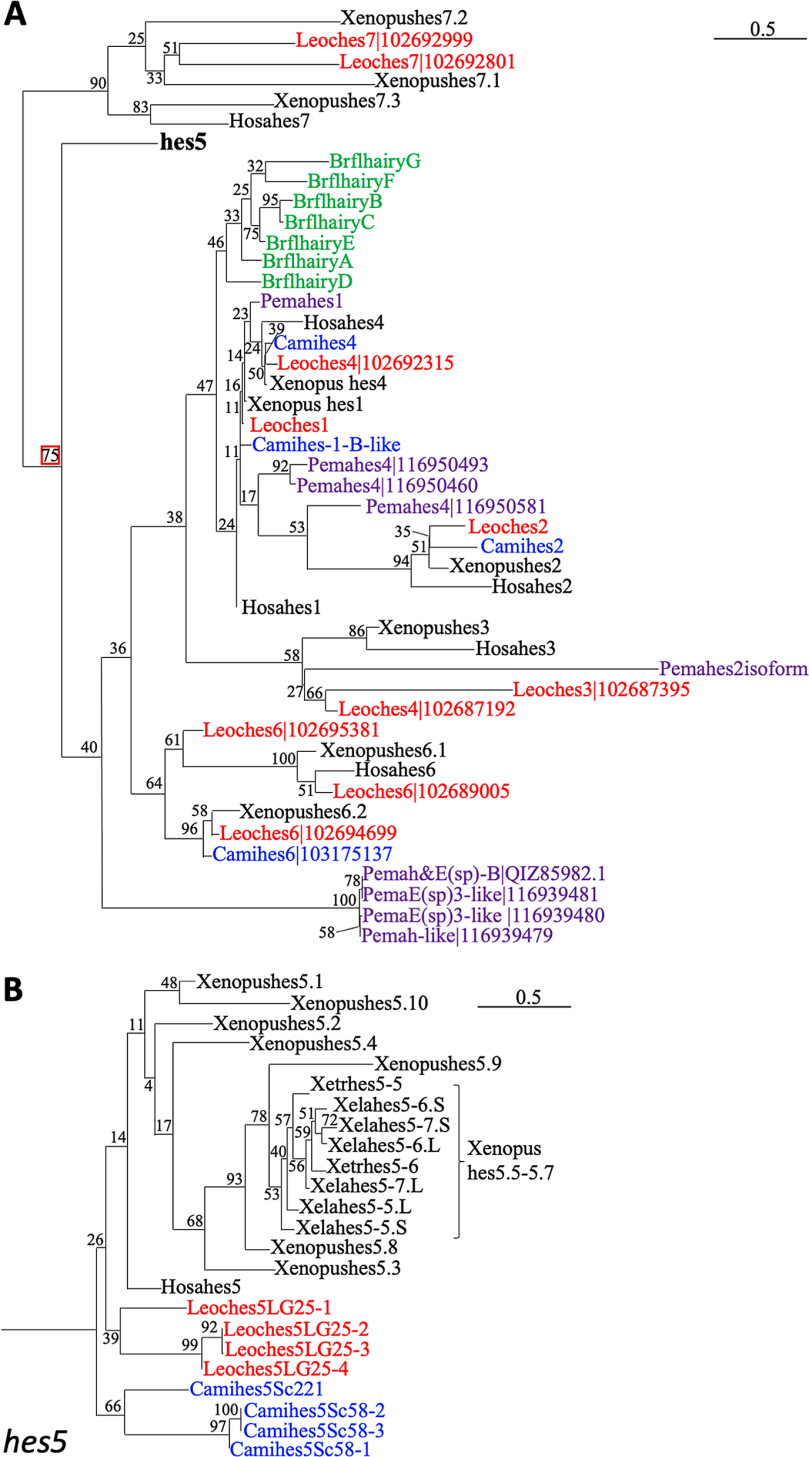
Phylogenetic analysis of *hes* genes of several jawed vertebrates. The phylogenetic tree was constructed by ML method. *hes* genes except for *hes5* (**A**), *hes5* genes (**B**) of spotted gar, elephant shark, lamprey and amphioxus. Blue, red, purple and green letters indicate spotted gar, elephant shark, lamprey and amphioxus, respectively. Hosa, human; Xetr, *Xenopus tropicalis*; Leoc, spotted gar; Cami, elephant shark; Pema, lamprey; Brfl, amphioxus

To determine the origin of the *hes5* cluster, we carried out phylogenetic analysis with spotted gar (*Lepisosteus oculatus*), elephant shark (*Callorhinchus milii*), lamprey (*Petromyzon marinus*), and amphioxus (*Branchiostoma floridae*) (Fig. 5A, B, Additional file 1: Fig. S3; the complete tree is shown in Additional file 1: Fig. S6). As a result, genes of *hes7* and *hes5* were clearly separated from the other genes with high bootstrap values. First, we counted the number of *hes* genes in these species except for *hes7*- and *hes5*-classified genes, although the bootstrap values were low. Spotted gar was suggested to have two *hes3*, two *hes7*, and three *hes6* (Fig. 5A, shown in red letter). Elephant shark had one *hes1, hes2, hes4,* and *hes6* (Fig. 5A, shown in blue letter). In lamprey, there were one *hes4*, three *hes2*, and one *hes3* (Fig. 5A, shown in purple letter). In amphioxus*, hairy A-G* genes were found, but were formed a single clade (Fig. 5A, shown in green letter).

In the *hes5* clade, both spotted gar and elephant shark possessed four genes (Fig. 5B), but all the genes were separately classified to the *Xenopus* genes (Fig. 5B, red and blue letters). From the results, we could not identify the homologous relationship of *hes5* genes between *Xenopus,* gar and elephant shark. In addition, no putative *hes5* genes were found in lamprey and amphioxus.

**Fig. 6:**
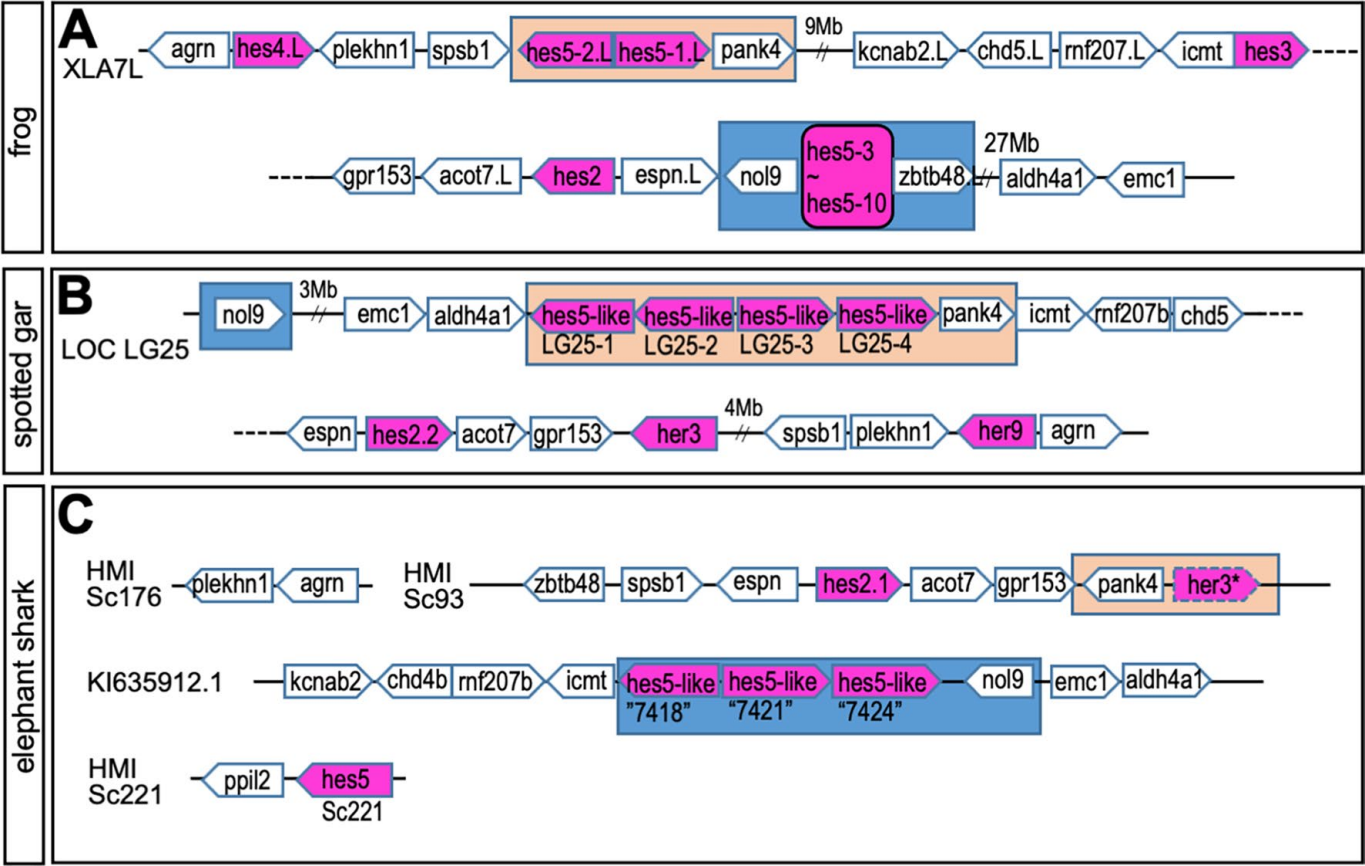
Comparison with *hes* gene locus among *Xenopus* (**A**), spotted gar (**B**) and elephant shark (**C**). Pentagon arrows show genes with the 5′–3′ direction. Magenta shows *hes* gene and magenta with a broken line is a pseudo gene. Orange square shows the *hes5-1* cluster region. Orange and blue square shows the *hes5.1* and *hes5.3* cluster region, respectively

Next, we compared the gene order on the genome around *Xenopus hes5* cluster locus in spotted gar and elephant shark. In linkage group (LG) 25 of the spotted gar, four *hes5-like* genes were located next to *pank4*, but no *hes5* (*-like*) genes were found near *nol9* (Fig. 6B)*.* This suggests that gar had a *hes5* cluster, and the cluster was closer to the *hes5.1* cluster than to the *hes5.3* cluster in *Xenopus*. In contrast, three of four *hes5* genes (*hes5-like*) in elephant shark were clustered near *nol9* on KI635912.1 (Fig. 6C). This suggests that the clustered genes might be related to the *hes5.3* cluster in *Xenopus*. In addition, the gene named *her3* was located near *pank4*, which is located near the *hes5.1* cluster in *Xenopus*, on HMISc93. Although the gene may have been given a wrong name because the sequence lacking WPRW domain, the synteny analysis suggests that the gene might be the homolog of *hes5*, and thus, a gene classified to the *hes5.1* cluster might be conserved in elephant shark. Another *hes5* gene in elephant shark was located next to *ppil2.* The order of the two genes was conserved in coelacanth (Fig. 2D), but not in *Xenopus*. One possible explanation for this is that the common ancestor of teleost and cartilaginous fishes had another *hes5* next to *ppil2,* but later lost the gene.

### Evolutionary phylogenetic relationships of *hes* gene in gnathostomes

**Fig. 7:**
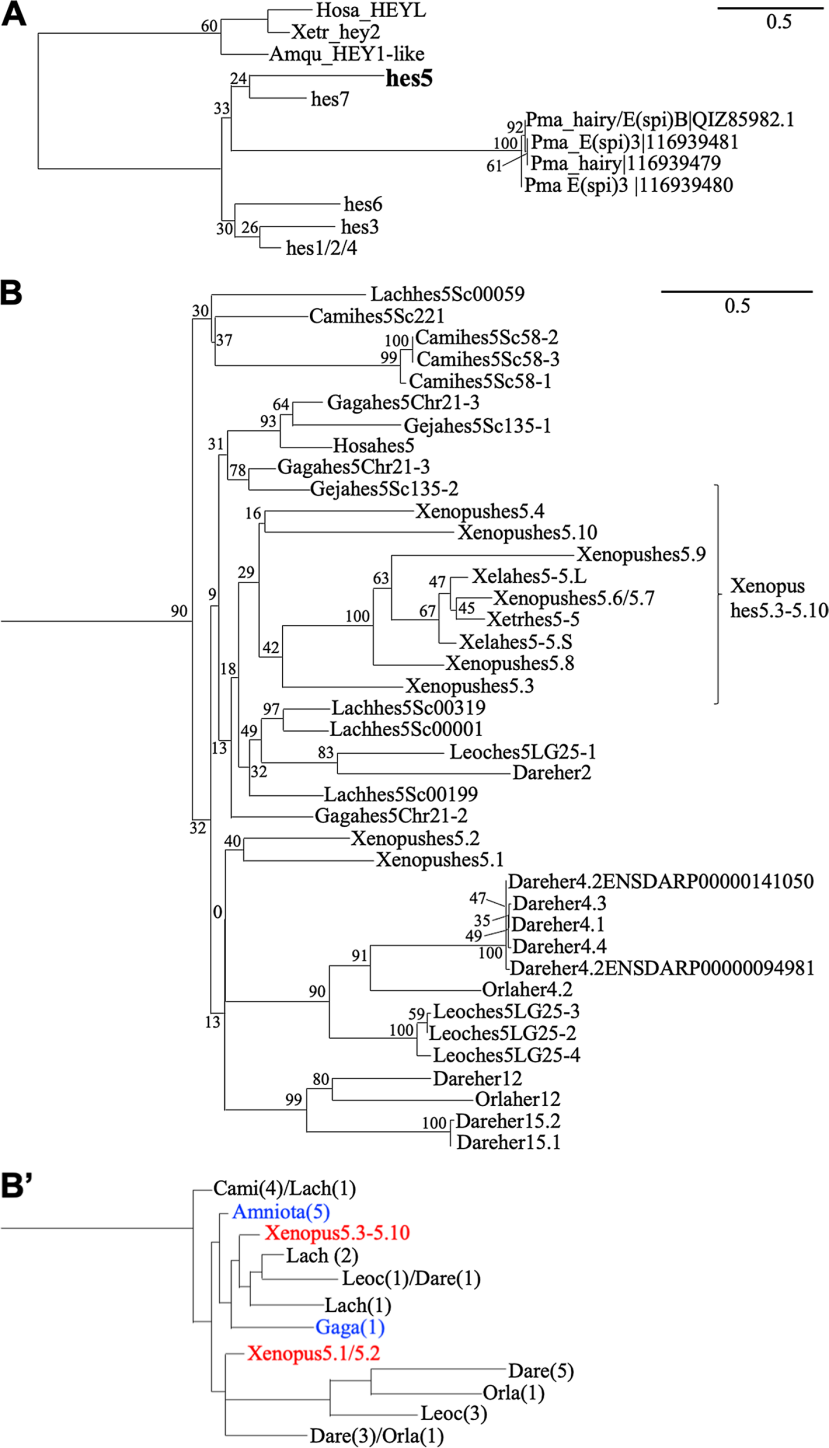
Comprehensive phylogenetic analysis of *hes genes* except for *hes5* (**A**) *and hes5 genes* (**B**) in jawed vertebrate. Evolutionary analysis was conducted in RAxML. Human HEYL, *X.tropicalis hey2* and sponge (*Amphimedon queenslandica*) HEY1-like gene sequences were used as an outgroup. The outline of **B** is described in **B’**. Hosa, human; Mumu, Mouse; Gaga, Chicken; Geja, Japanese gecko; Xetr, *Xenopus tropicalis*; Lach, Coelacanth; Dare, zebrafish; Orla, medaka; Leoc, spotted gar; Cami, elephant shark; Amqu, sponge

To further confirm the classification, we performed a phylogenetic analysis with human *hey* genes, which are reported to be close to *hes* genes, as an outgroup (Fig. 7A: complete tree is shown in Additional file 1: Fig. S7) [17]. As it can be seen from the low bootstrap values in the *hes* clades, the phylogenetic tree was not solved well. However, as we already discussed above, eight zebrafish genes (*her4.1-4.4, her12, her15.1-15.2*), two medaka genes (*Orlaher4.2, 12*), and three gar genes formed a single clade with *Xenopus hes5.1-5.2* (Fig. 7B, B’). However, a monophyletic group including *Xenopus hes5.3-5.10*, three coelacanth *hes5* (LachSc00001, 00119, 00319), Zebrafish *her2,* and one gar *hes5* (*LeocLG25-1*) was formed (Fig. 7B, B’), even though *LachSc00319* and *LeocLG25-1* showed syntenic similarity with the *hes5.1* cluster (Figs. 2D, 6B).

**Fig. 8:**
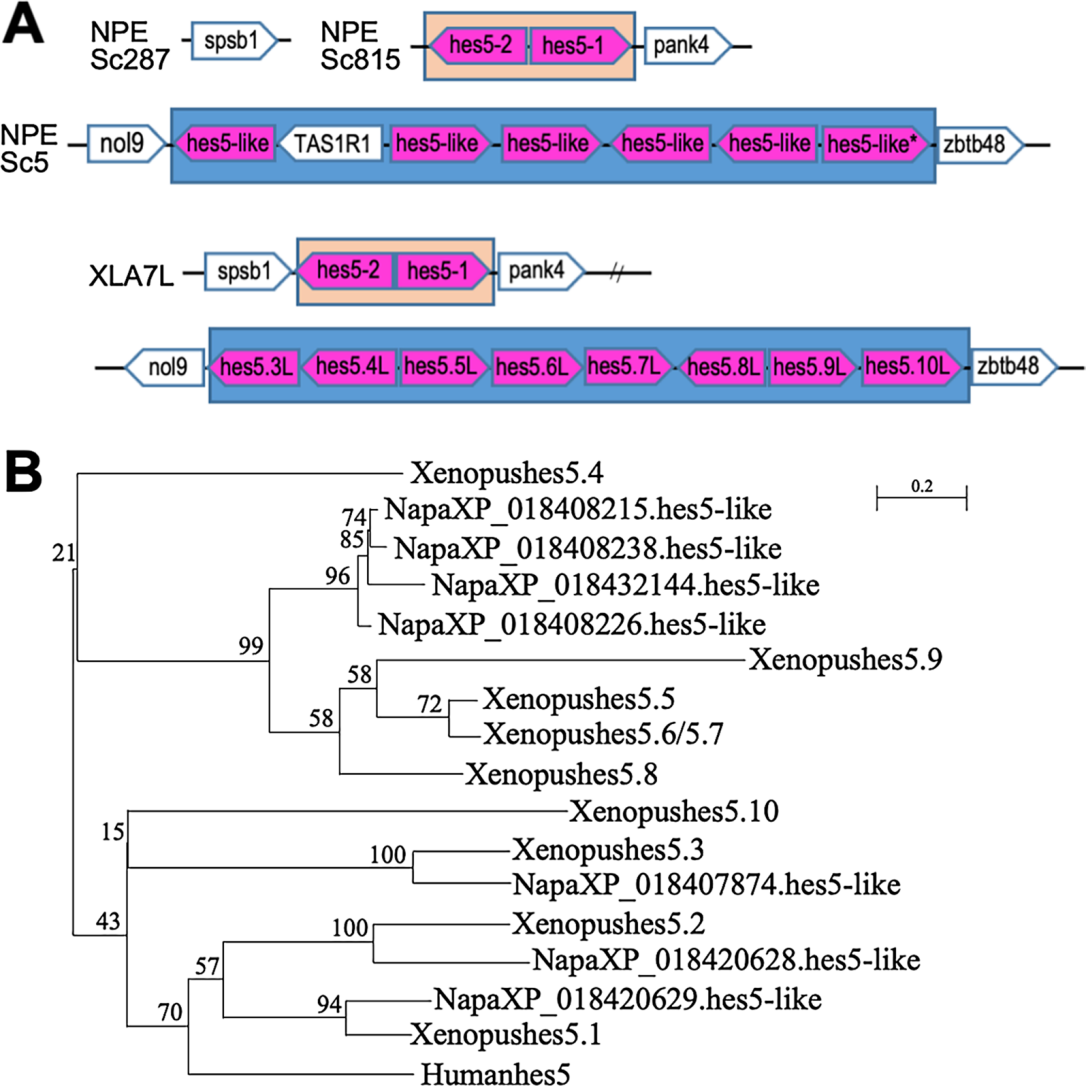
Syntenic and phylogenetic analysis of Tibetan frog *hes5* genes. **A** Synteny analysis around *hes5* locus in *Xenopus laevis* and *Nanorana parkeri.* *: the gene lacks WPRW motif (the gene was deleted from the following phylogenetic analysis (**B**)). **B** Phylogenetic tree analysis of *hes5* genes of *Xenopus laevis, Xenopus tropicalis* and *Nanorana parkeri*. The phylogenetic tree was constructed by ML method

Since two *hes5* clusters with a high number of genes was conserved in two *Xenopus* species, we next examined the possibility of conservation in frogs. To determine this, we analyzed another frog species, Tibetan frog (*Nanorana parkeri*). From the synteny analysis, many *hes5-like* genes were found to be clustered on the genomes: two *hes5-like* genes were located next to *pank4*, and six *hes5-like* genes were located between *nol9* and *zbtb48* (although *TAS1R1* was inserted into the *hes* gene cluster locus, which was not found around the *hes5.1* or *hes5.3* cluster in *Xenopus*) (Fig. 8A). These results suggest that the two *hes5* genes on Scaffold815 of Tibetan frog are classified to the *hes5.1* cluster and the other six genes on Scaffold5 are to the *hes5.3* cluster. Consistently, phylogenetic analysis showed that two Tibetan frog *hes5* formed a single clade with the *hes5.1* cluster genes of *Xenopus* and the other Tibetan frog *hes5* genes formed a clade with *Xenopus hes5.4*-*5.9*, the *hes5.3* cluster genes (Fig. 8B: complete tree was shown in Additional file 1: Fig. S8). These results suggest that the two *hes5* clusters are conserved among frogs.

## Discussion

**Fig. 9:**
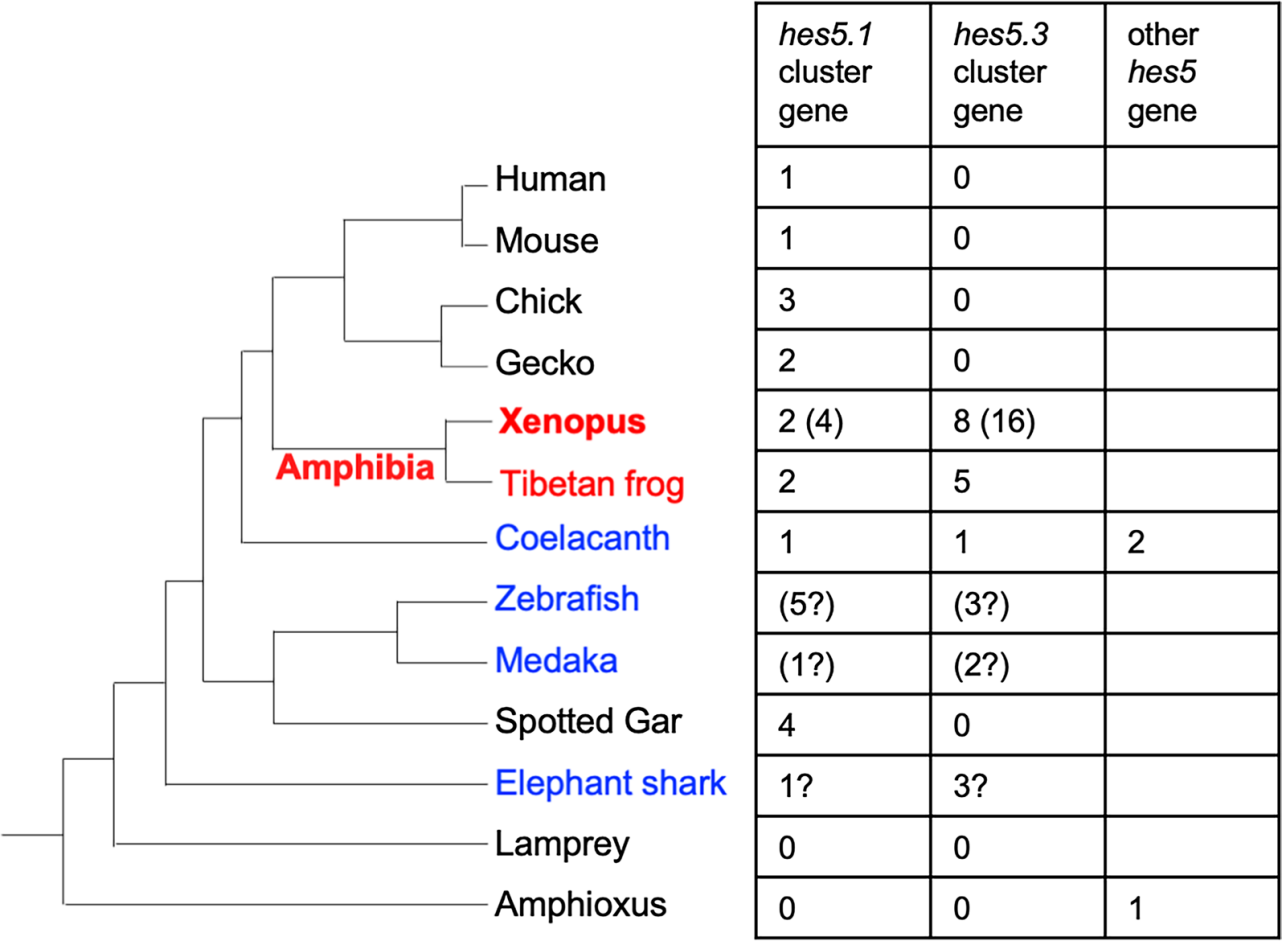
Evolutionary acquisition of *hes5* genes and the *hes5* cluster. Phylogenetic tree of jawed vertebrate (left). The table shows the number of *hes5* genes that were classified to the *hes5.1* or *hes5.3* cluster and the number of *hes5* genes that were not classified to the two clusters. The phylogenetic analysis was conducted using full-length amino acid sequences

Our study showed that *hes5* gene was absent in lamprey and amphioxus, but was existed in Gnathostomata, suggesting that the gene was acquired at the common ancestor of Gnathostomata (Figs. 5, 9). However, there are still other possibilities. Eight *hairy* genes have been reported in amphioxus, at least four of which have conserved gene expression patterns in vertebrates (in the central nervous system, presomitic mesoderm, somites, notochord, and gut) [18]. Considering from this, it is also possible that other *hes* genes (*hairy*) substitute for *hes5* function in these species.

We found that elephant shark possessed *hes5* (Figs. 5B, 9). Interestingly, synteny analysis indicated that three *hes5* genes might be the orthologue of the *hes5.3* cluster in *Xenopus* (Fig. 6C). In addition, a putative *hes5.1* cluster gene, which is named as *her3*, existed near *pank4* in the shark (Fig. 6C). In contrast, no *hes5*-related gene was found in amphioxus and lamprey (Fig. 9). These results suggest that a common ancestor of gnathostomata acquired both *hes5.1* and *hes5.3* genes.

In spotted gar, we could not identify any genes that belong to the *hes5.3* cluster, although we could find many *hes5-like* genes, which belong to the *hes5.1* cluster (Figs. 6B, 9). We wonder how it is evolved: one possible explanation is that, when the ancestor evolved into cartilaginous fishes and neopterygii, the genes called three *hes5-like* genes in the shark near *nol9* (the *hes5.3* cluster) was translocated to the locus next to *pank4* and partially duplicated. Another possibility is that *her3* (the *hes5.1* cluster) was duplicated, and three *hes5-like* genes (the *hes5.3* cluster) in elephant shark were lost in the spotted gar. Unfortunately, we could not obtain direct evidence for these possibilities from phylogenetic analysis (Fig. 5).

The *hes5.1* and *hes5.3* cluster seemed to be conserved in both teleost and neopterygian (Figs. 4, 6). On the other hand, although many *hes5* genes were existed in coelacanths (Fig. 2), no cluster was found.From this point of view, what is characteristic of coelacanths is the presence of *hes5*, in which only the synteny of elephant shark is preserved, so the evolution of *hes5* is different between sarcopterygians and cartilaginous fishes, and only sarcopterygians and cartilaginous fishes seem to have preserved hes5 differently from other animals. Although further analysis for the connection of the scaffold is needed, the genes in the coelacanth are possibly the prototype of the *hes5.1* and *hes5.3* cluster genes. All amniote *hes5* genes seemed to be classified to the *hes5.1* cluster, and not the *hes5.3* cluster (Figs. 1, 2, 9), suggesting that the *hes5.3* clusters was lost after branching into amniotes.

We further performed gene structure analysis: the number of exons in the coding regions of each *hes5* gene as Zhou et al. [17]. In both *X. tropicalis* and *X. laevis,* almost all *hes5* consisted of three exons, except for *hes5.8*. On the other hand, *hes5* genes of many actinopterygian including zebrafish, medaka, and spotted gar genes possessed two exons in coding region (Table 2). This might reflect that *hes5* genes in actinopteryozoa and osteichthyes were increased in an independent manner.

**Table 2:** The number of exon, amino acids, and the synteny similarity

	The number of exons	The number of amino acids	Synteny similar to
*Human hes5*	2	166aa	*hes5.1*
*Mouse hes5*	2	X1 167aa, X2 141aa	*hes5.1*
*Chicken hes5 chr21-1*	5	161aa	*hes5.1*
*Chicken hes5 chr21-2*	3	197aa	*hes5.1*
*Chicken hes5 chr21-3*	3	157aa	*hes5.1*
*Gekko japonicus hes5 sc135-1*	2	161aa	*hes5.1*
*Gekko japonicus hes5 sc135-2*	3	157aa	*hes5.1*
*Xenopus tropicalis hes5.1*	3	154aa	*hes5.1*
*Xenopus laevis hes5.1L*	3	154aa	*hes5.1*
*Xenopus laevis hes5.1S*	3	154aa	*hes5.1*
*Xenopus tropicalis hes5.2*	3	158aa	*hes5.1*
*Xenopus laevis hes5.2L*	3	158aa	*hes5.1*
*Xenopus laevis hes5.2S*	3	159aa	*hes5.1*
*Xenopus tropicalis hes5.3*	3	160aa	*hes5.3*
*Xenopus laevis hes5.3L*	3	183aa	*hes5.3*
*Xenopus laevis hes5.3S*	3	164aa	*hes5.3*
*Xenopus tropicalis hes5.4*	3	159aa	*hes5.3*
*Xenopus laevis hes5.4L*	3	159aa	*hes5.3*
*Xenopus laevis hes5.4S*	3	159aa	*hes5.3*
*Xenopus tropicalis hes5.5*	3	158aa	*hes5.3*
*Xenopus tropicalis hes5.5*	3	158aa	*hes5.3*
*Xenopus laevis hes5.5L*	3	158aa	*hes5.3*
*Xenopus laevis hes5.5S*	3	150aa	*hes5.3*
*Xenopus tropicalis hes5.6*	3	148aa	*hes5.3*
*Xenopus laevis hes5.6L*	3	156aa	*hes5.3*
*Xenopus laevis hes5.6S*	3	156aa	*hes5.3*
*Xenopus laevis hes5.7L*	3	154aa	*hes5.3*
*Xenopus laevis hes5.7S*	3	X1 155aa, X2 154aa	*hes5.3*
*Xenopus tropicalis hes5.8*	4	X1 145aa. X2 141aa	*hes5.3*
*Xenopus laevis hes5.8L*	3	140aa	*hes5.3*
*Xenopus laevis hes5.8S*	3	149aa	*hes5.3*
*Xenopus tropicalis hes5.9*	3	155aa	*hes5.3*
*Xenopus laevis hes5.9L*	3	155aa	*hes5.3*
*Xenopus laevis hes5.9S*	3	155aa	*hes5.3*
*Xenopus tropicalis hes5.10*	3	166aa	*hes5.3*
*Xenopus laevis hes5.10L*	3	144aa	*hes5.3*
*Xenopus laevis hes5.10S*	3	166aa	*hes5.3*
*coelacanth hes5 sc00319*	3	164aa	*hes5.1*
*coelacanth hes5 sc00199*	2	135aa	*hes5.3*
*coelacanth hes5 sc00059*	2	146aa	shark Sc221, Xenopus chromosome1
*coelacanth hes5 sc00001*	3	190aa	*Xenopus* chromosome 7
*zebrafish her4.1*	2	152aa	*hes5.1?*
*zebrafish her4.2 ENSDARP00000141050*	2	152aa	*hes5.1?*
*zebrafish her4.3*	2	152aa	*hes5.1?*
*zebrafish her4.4*	2	152aa	*hes5.1?*
*zebrafish her4.2 ENSDARP00000094981*	2	152aa	*hes5.1?*
*zebrafish her12*	2	155aa	*hes5.1?*
*zebrafish her2*	2	108aa	*hes5.3?*
*zebrafish her15.1*	2	149aa	*hes5.3?*
*zebrafish her15.2*	2	149aa	*hes5.3?*
*medaka her4.2*	2	166aa	*hes5.1? hes5.3?*
*medaka her12*	2	145aa	*hes5.1? hes5.3?*
*spotted gar hes5 LG25-1*	2	163aa	*hes5.1*
*spotted gar hes5 LG25-2*	3	159aa	*hes5.1*
*spotted gar hes5 LG25-3*	2	159aa	*hes5.1*
*spotted gar hes5 LG25-4*	2	162aa	*hes5.1*
*elephant shark hes5 Sc58-1*	3	169aa	*hes5 3*
*elephant shark hes5 Sc58-2*	3	169aa	*hes5.3*
*elephant shark hes5 Sc58-3*	3	169aa	*hes5.3*
*elephant shark hes5 Sc221*	8	475aa	coelacanth *hes5* sc00059, Xenopus chromosome 1

The gene names in the vertical direction. X1 or X2 means the variants

The number of *hes5* genes is specifically high in frogs, especially the *hes5.3* cluster genes. To estimate the duplication process, comparison of the transcriptional direction is considered important [11]. As we previously reported, the directions of *hes5.5, 5.6, 5.7*, and *5.9* are same. Phylogenetic analysis also indicated that these genes were closely mapped in the tree (Fig. 1A), suggesting that these genes may share a common origin, and may be tandemly duplicated in *Xenopus*. Phylogenetic analysis also indicated that *hes5.1, hes5.2*, and *hes5.10* showed high similarity (Fig. 1B, 3B, 5B, and 7B). This result suggests another possibility that *hes5.10* duplicated from *hes5.1*/*5.2*.

In general, *hes5* functions downstream of Notch signaling and inhibits neuronal differentiation [19, 20]. RNA-seq analysis revealed that the expression of almost all *hes5* genes is high during the gastrula and neurula stages, at which Notch signaling is activated, in *Xenopus* [11]. These results imply that the function of *hes5* is possibly conserved between mouse and *Xenopus*. Thus, how these duplicated *hes5* genes work in neurogenesis remains to be investigated, and this may elucidate the significance of the higher number of *hes5* genes in the frogs.

## Conclusions

In this study, to reveal the evolutionary process of *hes* genes, we elucidatedthe orthologous relationship of *hes* genes in vertebrates using phylogenetic and synteny analyses. In addition, we estimated the evolutionary origins of the two *hes5* clusters, which have been found in *Xenopus*. Although *hes5* genes were found in other jawed vertebrates, the number of *hes5* genes was higher in frogs. The rudiment of the two clusters was found in elephant shark, suggesting that ancestral species of chondrichthyans might have the origin of these clusters. These findings go a step further in the research on the function of all *hes* genes in vertebrates as well as the understanding of the evolutionary process of large gene clusters.

## Methods

### Protein sequencing comparison

A multiple alignment of protein sequence of *hes* genes were visualized with MUSCLE [21].

### Phylogenetic analysis

Phylogenetic analysis was performed using RAxML (v8.2.0) [22]. Multiple alignments of protein sequence were carried out using MAFFT (v7.221) [23] with the auto strategy. Unaligned regions were trimmed with TrimAl (v1.2rev59) [24] using the gappyout option and phylogeny trees were constructed by the maximum likelihood method with PROTGAMMAAUTO.

### Annotation of genes: GenBank accessions of *hes* genes

Genomic synteny of *hes* genes was analyzed using genome assemblies of *X. laevis* (v9.1), *X. tropicalis* (v9) from *Xenopus* genome project (http://viewer.shigen.info/Xenopus/).

Other species Gene ID and accession number for these analyses are from NCBI (https://www.ncbi.nlm.nih.gov/) and Ensembl (https://asia.ensembl.org/index.html).

*hes5* genes are as follows: *hes*5chr21-1 ID: 419390. *hes*5chr21-2 ID: 107057363. *hes*5chr21-3 ID: 419,392. *hes*5sc135-1 ID: 107122264. *hes*5Sc135-2 ID: 107122267. Lach *hes*5Sc00059 ID: 102346872. *hes*5Sc00199 ID: 102346203. Lepisosteus oculatus *hes*5 LG25-1 ID: 102684766. *hes*5LG25-2 ID: 102684967. *hes*5 LG25-3 ID: 102683774. *hes*5LG25-4 ID: 102685165. Callorhinchus milii *hes*5Sc221 ID: 103188596. *hes*5Sc58-2 ID: 103181452. *hes*5Sc58-3 ID: 103181453. *hes*5Sc58-1 ID: 103181414.

## Supplementary Information

Additional file 1: **Table S1.** The list of *hes* genes in *Danio rerio* and *Oryzias latipes*. **Figure S1.** Syntenic comparison with *hes1, 6* and *hes7* gene loci. **Figure S2.** Multiple alignment of amino acid sequences of *hes5*. **Figure S3.** Multiple alignment of amino acid sequence of lamprey (Pema) and amphioxus (Brfl) *hes* genes. **Figure S4.** Detail phylogenetic tree of *hes* genes in sarcopterygian. **Figure S5.** Detail phylogenetic tree of *hes* genes in teleosts. **Figure S6.** Detail phylogenetic tree of *hes* genes in jawed vertebrates. **Figure S7.** Detail phylogenetic tree of all *hes5* genes we observed. **Figure S8.** Detail phylogenetic tree of all *hes5* genes in Tibetan frog.

## Data Availability

All data is presented within the manuscript including supplemental materials.
