# Supplementary material for: Evolution of hes gene family in vertebrates: the hes5 cluster genes have specifically increased in frogs
Source: BMC Ecol Evol. 2021 Jul 29;21:147. doi: 10.1186/s12862-021-01879-6 (PMC8320183; doi:10.1186/s12862-021-01879-6)
Supplement: Supplementary file 1 — Additional file 1: Table S1. The list of hes genes in Danio rerio and Oryzias latipes. Figure S1. Syntenic comparison with hes1, 6 and hes7 gene loci. Figure S2. Multiple alignment of amino acid sequences of hes5. Figure S3. Multiple alignment of amino acid sequence of lamprey (Pema) and amphioxus (Brfl) hes genes. Figure S4. Detail phylogenetic tree of hes genes in sarcopterygian. Figure S5. Detail phylogenetic tree of hes genes in teleosts. Figure S6. Detail phylogenetic tree of hes genes in jawed vertebrates. Figure S7. Detail phylogenetic tree of all hes5 genes we observed. Figure S8. Detail phylogenetic tree of all hes5 genes in Tibetan frog. [file 12862_2021_1879_MOESM1_ESM.pdf]

Evolution of *hes* gene family in vertebrates: the *hes5* cluster genes have specifically increased in frogs

Figure legends for additional materials

Table S1. The list of *hes* genes in *Danio rerio* and *Oryzias latipes*.

Fig. S1. Syntenic comparison with *hes1, 6* and *hes7* gene loci

(A) Comparison of *hes1, 6* loci among *Xenopus*, human and chicken. Paired curved blue arrows indicate flipping of genome sequences to align gene orders. A broken line circle indicates no gene model but the existence of homologous gene sequence, implying that the genome sequence is not complete or this might be a pseudogene. Pentagon arrows show genes with 5' to 3' direction. **(B)** Comparison of *hes1, 6* loci among *Xenopus* and zebrafish. **(C)** Comparison with *hes7* gene genomic regions among *Xenopus*, human and chicken. **(D)** Comparison with *hes7* gene genomic regions between *Xenopus* and zebrafish. HSA, human; GGA, chicken; XTR, *Xenopus tropicalis*; XLA, *Xenopus laevis*; DRE, zebrafish.

Fig. S2. Multiple alignment of amino acid sequences of *hes5*

Multiple alignment of amino acid sequence of *hes5* was made by MUSCLE. Domain names and their boundaries are shown below the alignments. Asterisk show the conserved amino acid residues. Conserved Proline (P) residues are shown by red frames. Xela, *Xenopus laevis*; Xetr, *Xenopus tropicalis*; Hosa, human; Mumu, mouse; Gaga, chicken; Lach, coelacanth; Leoc, spotted gar; Dare, zebrafish; Orla, medaka; Cami, elephant shark.

Fig. S3. Multiple alignment of amino acid sequence of lamprey (Pema) and amphioxus (Brfl) *hes* genes

Domain names and their boundaries are shown below the alignments. Asterisk show the conserved amino acid residues. Conserved Proline (P) residues are shown by red frame.

Fig. S4. Detail phylogenetic tree of *hes* genes in sarcopterygian

Fig. S5. Detail phylogenetic tree of *hes* genes in teleosts

Fig. S6. Detail phylogenetic tree of *hes* genes in jawed vertebrates

Fig. S7. Detail phylogenetic tree of all *hes5* genes we observed

Fig. S8. Detail phylogenetic tree of all *hes5* genes in Tibetan frog

zebrafish gene name	Synonym (with detail information)	position	Xenopus gene
her12	Hes5c	Chromosome 23 reverse strand.	hes5
her4.2	fe18g04, Hes5d, si:ch73-21g5.3	Chromosome 23 reverse strand.	hes5
her4.4	si:ch73-21g5.5	Chromosome 23 reverse strand.	hes5
her9	Hes1a, wu:fc30a03, zgc:100766	Chromosome 23 reverse strand.	hes5
her4.3	si:ch73-21g5.4, wu:fe26g04	Chromosome 23 forward strand.	hes5
her4.1	ch73-21g5.2, her4, Hes5e, wu:fe18g04, wu:fq41c06, zgc:55218	Chromosome 23 reverse strand.	hes5
her4.2	fe18g04, Hes5d, si:ch73-21g5.3	Chromosome 23 forward strand.	hes5
her15.1	fk51a02, her15, her15a, hes5, wu:fk51a02, zgc:136520	Chromosome 11 forward strand.	hes5
her15.2	her15, her15b, hes5, hes5-like	Chromosome 11 reverse strand.	hes5
her2	Hes5a	Chromosome 11 reverse strand.	hes5
her3	hes3	Chromosome 8 forward strand.	hes3
her11	him	Chromosome 14 reverse strand.	hes7-3
her5	zgc:136537	Chromosome 14 forward strand.	hes7 (7-1)
her8.2	Hes8	Chromosome 15 forward strand.	hes6-2
her13	her11, her13.1, hes6b, zgc:110599	Chromosome 15 forward strand.	hes6-1
her1	etID309753.5, Hes7, id:ibd5086	Chromosome 5 reverse strand.	hes7-3
her7	cb715	Chromosome 5 forward strand.	hes7 (7-1)
her6	cb129, Hes1b	Chromosome 6 reverse strand.	hes1
her8a	her8b, wu:fb75f05, wu:fc06c04, zgc:85712	Chromosome 7 reverse strand.	hes6-2
hes6	her13.2	Chromosome 2 forward strand.	hes6
hes2.1	Hes2a, si:ch211-122n14.3	Chromosome 8 reverse strand.	hes2
hes2.2	Hes2b, zgc:153398	Chromosome 8 reverse strand.	hes2
medaka gene name	Synonym (with detail information)	position	Xenopus gene
her9	Hes4, Hes1a, wu:fc30a03, zgc:100766	Chromosome 7 forward strand.	(hes4)
her12	Hes5c	Chromosome 7 reverse strand.	hes5
her4.4	si:ch73-21g5.5	Chromosome 7 reverse strand.	hes5
hes2.2	Hes2b, zgc:153398	Chromosome 7 forward strand.	hes2
hes7	Oryzias latipes Her1-11 (LOC100049416) [Source: RefSeq mRNA; Acc: NM_001104797]	Chromosome 10 forward strand.	hes7-3
her5	zgc:136537	Chromosome 10 reverse strand.	hes7 (7-1)
her6.2	Oryzias latipes Her6.2 (LOC100049440) [Source:RefSeq mRNA; Acc: NM_001104814]	Chromosome 17 reverse strand.	(hes1)
hes6 (2of2)	bHLHb41	Chromosome 17 reverse strand.	hes6-1
her8.2	Hes8	Chromosome 13 forward strand.	hes6-2
hes6	Her13.2 [Source:RefSeq peptide; Acc: NP_001098282]	Chromosome 13 forward strand.	hes6-1
her7	cb715	Chromosome 16 reverse strand.	hes7 (7-1)
her6	cb129, Hes1b	Chromosome 4 reverse strand.	hes1

Sup.Table1

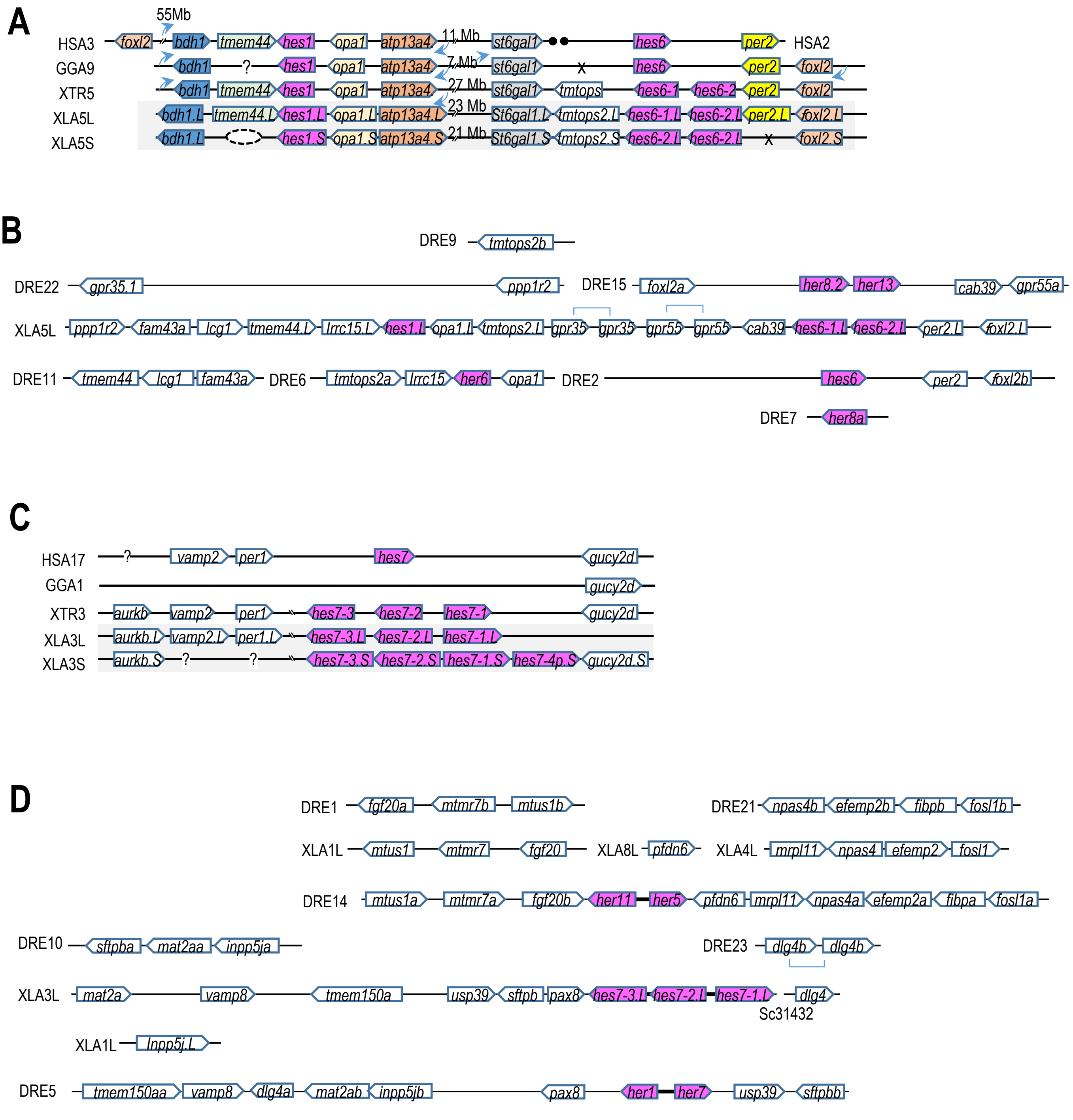
A
55Mb
HSA3
foxl2
bdh1
tmem44
hes1
opa1
atp13a4
11 Mb
st6gal1
hes6
per2
HSA2
GGA9
7 Mb
foxl2
XTR5
27 Mb
tmtops
hes6-1
hes6-2
XLA5L
bdh1.L
tmem44.L
hes1.L
opa1.L
atp13a4.L
23 Mb
St6gal1.L
tmtops2.L
hes6-1.L
hes6-2.L
per2.L
foxl2.L
XLA5S
hes1.S
opa1.S
atp13a4.S
21 Mb
St6gal1.S
tmtops2.S
foxl2.S
B
DRE9
tmtops2b
DRE22
gpr35.1
ppp1r2
DRE15
foxl2a
her8.2
her13
cab39
gpr55a
XLA5L
fam43a
lcg1
lrrc15.L
gpr35
gpr55
DRE11
tmem44
fam43a
DRE6
tmtops2a
lrrc15
her6
DRE2
foxl2b
DRE7
her8a
C
HSA17
vamp2
per1
hes7
gucy2d
GGA1
XTR3
aurkb
hes7-3
hes7-2
hes7-1
XLA3L
aurkb.L
vamp2.L
per1.L
hes7-3.L
hes7-2.L
hes7-1.L
XLA3S
aurkb.S
hes7-3.S
hes7-2.S
hes7-1.S
hes7-4p.S
gucy2d.S
D
DRE1
fgf20a
mtmr7b
mtus1b
DRE21
npas4b
efemp2b
fibpb
fosl1b
XLA1L
mtus1
mtmr7
fgf20
XLA8L
pfdn6
XLA4L
mrpl11
npas4
efemp2
fosl1
DRE14
mtus1a
mtmr7a
fgf20b
her11
her5
fibpa
fosl1a
DRE10
sftpba
mat2aa
inpp5ja
DRE23
dlg4b
XLA3L
mat2a
vamp8
tmem150a
usp39
sftpb
pax8
dlg4
Sc31432
XLA1L
Inpp5j.L
DRE5
tmem150aa
dlg4a
mat2ab
inpp5jb
her1
her7
sftpbb

Fig. S1

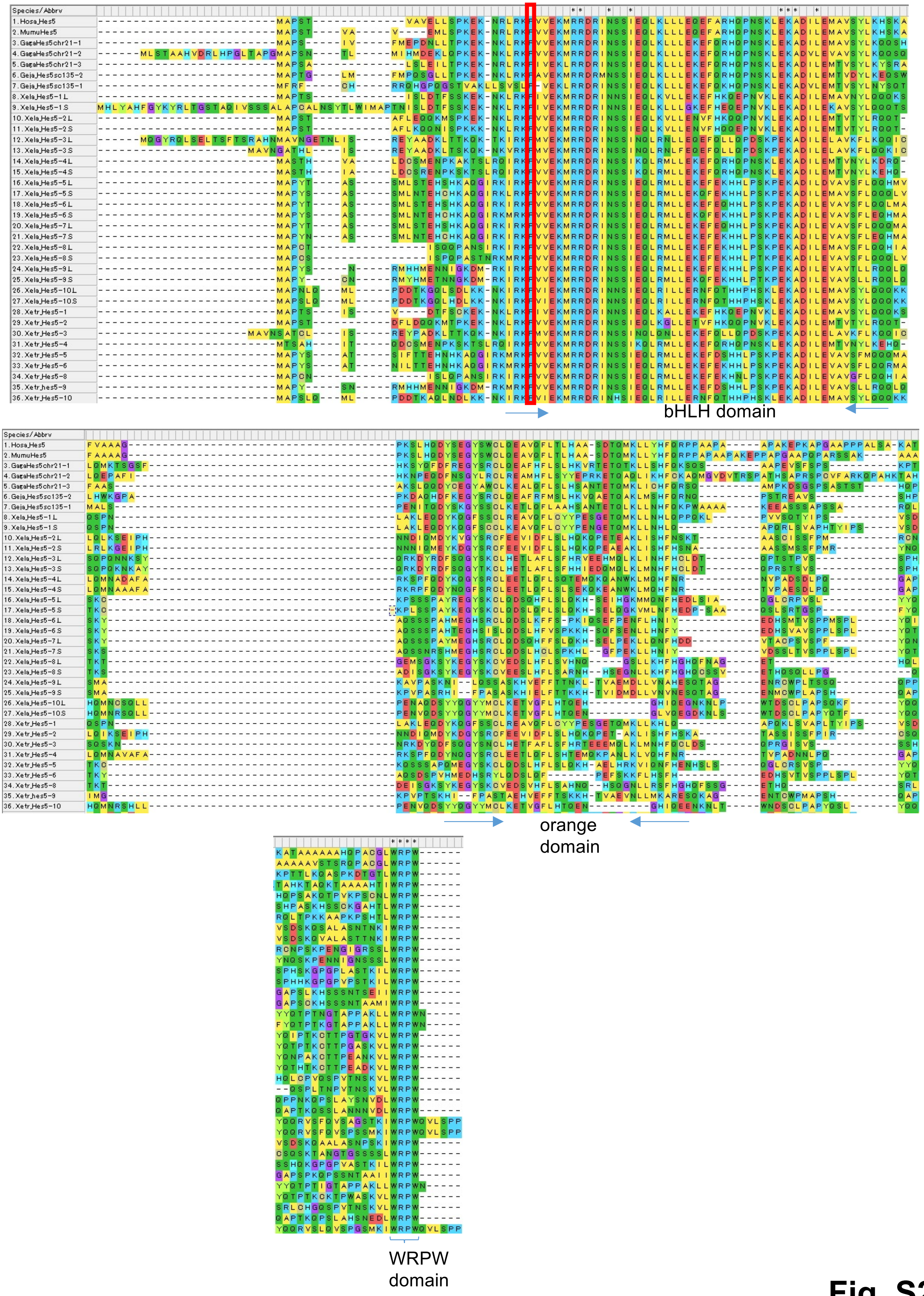

Fig. S2

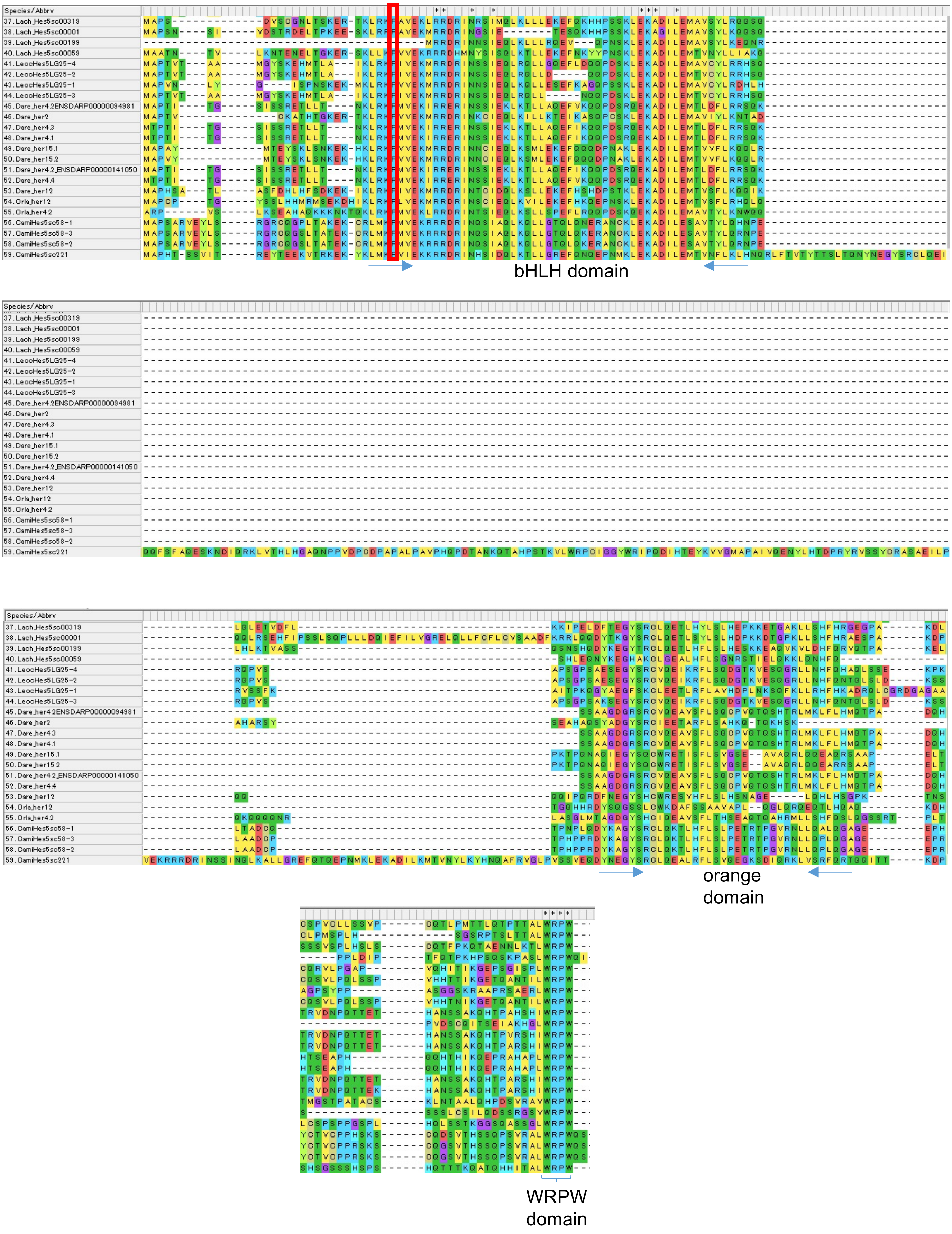

Fig. S2 (continued)

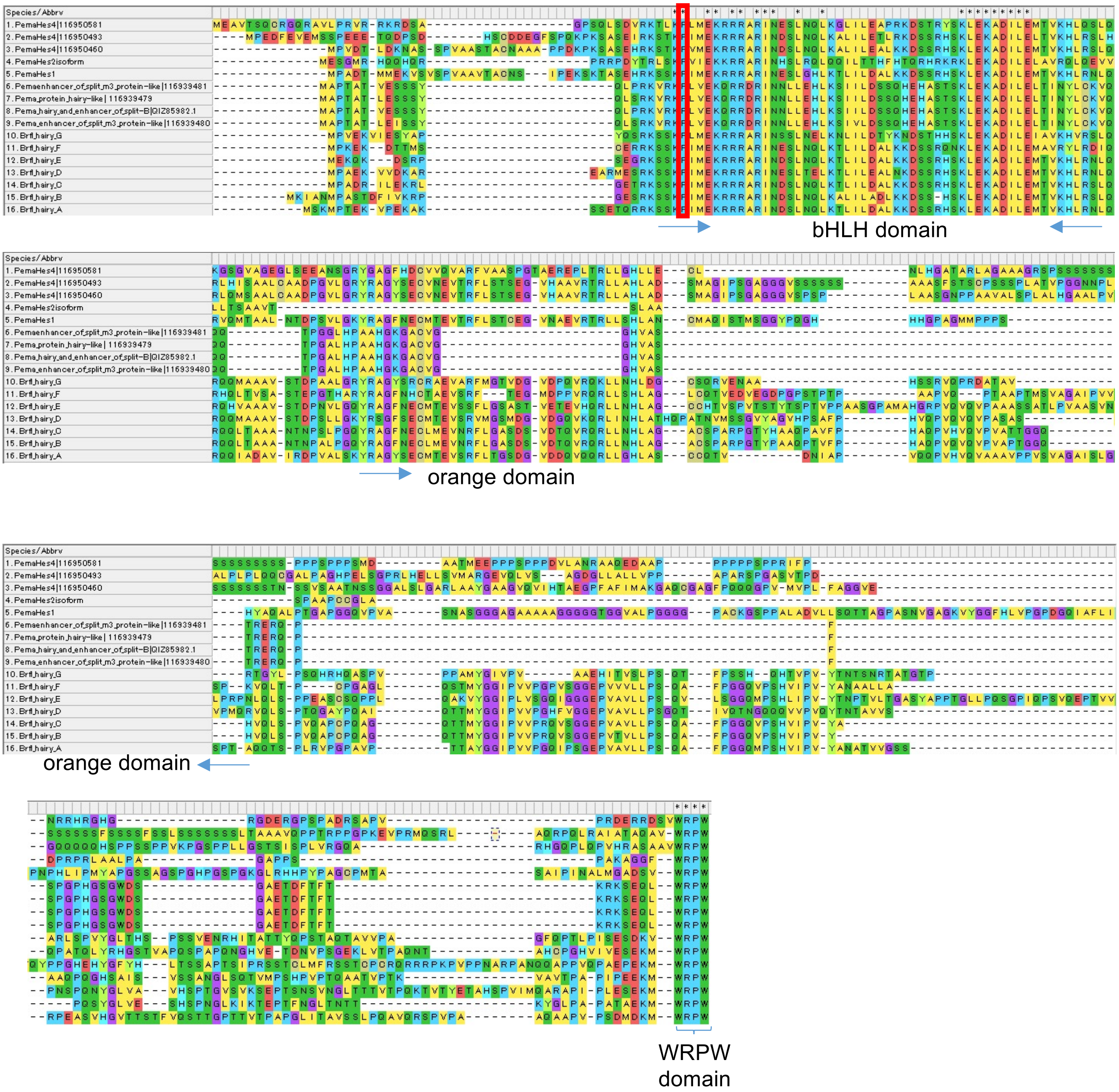
Species/Abbrv
1. PemaHes4|116950581
2. PemaHes4|116950493
3. PemaHes4|116950460
4. PemaHes2isoform
5. PemaHes1
6. Pemaenhancer_of_split_m3_protein-like|116939481
7. Pema_protein_hairy-like|116939479
8. Pema_hairy_and_enhancer_of_split-B|QIZ85982.1
9. Pema_enhancer_of_split_m3_protein-like|116939480
10. Brfl_hairy_G
11. Brfl_hairy_F
12. Brfl_hairy_E
13. Brfl_hairy_D
14. Brfl_hairy_C
15. Brfl_hairy_B
16. Brfl_hairy_A
bHLH domain
orange domain
orange domain
WRPW
domain

Fig. S3

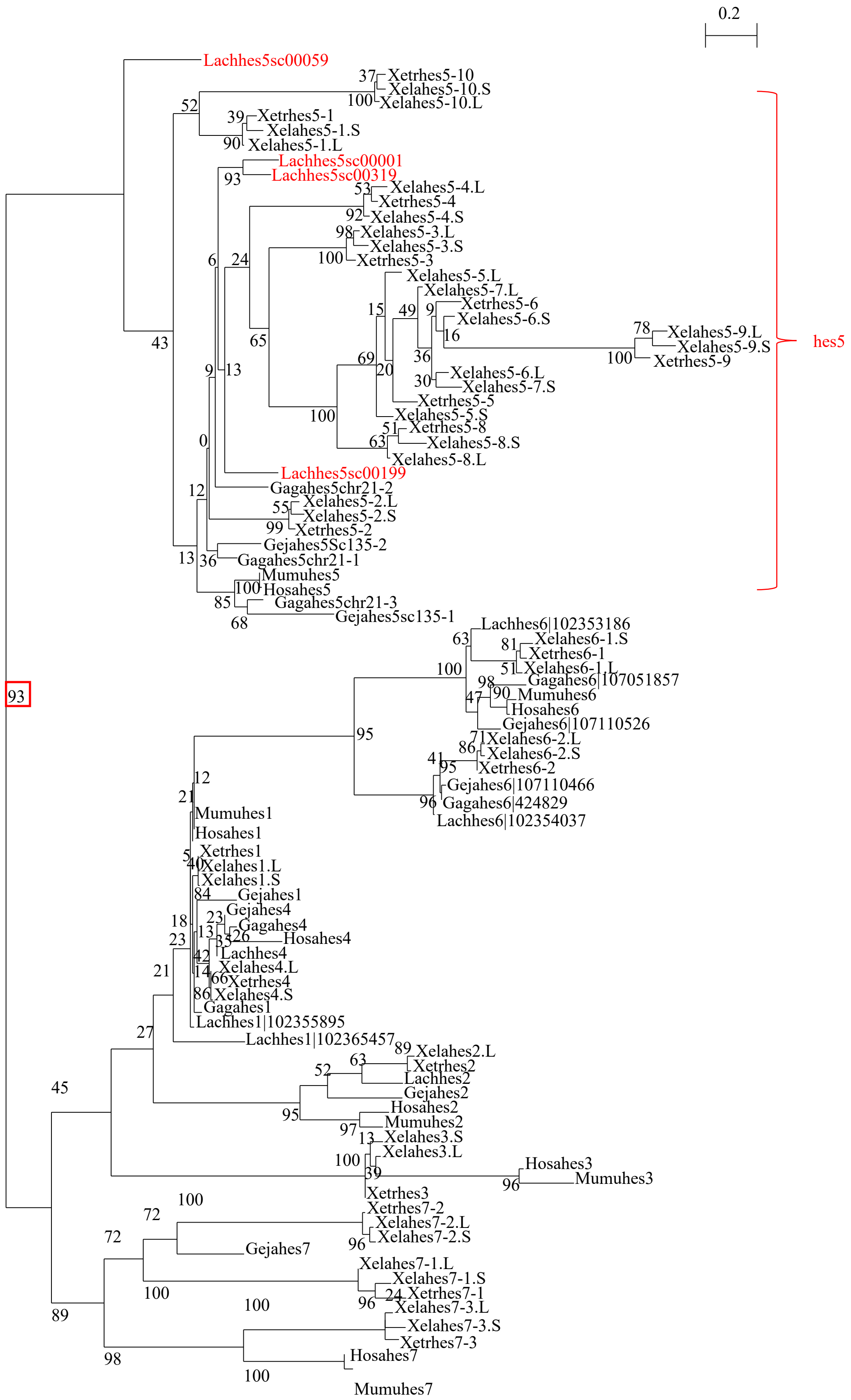

0.2
Lachhes5sc00059
Xetrhes5-10
Xelahes5-10.S
Xelahes5-10.L
Xetrhes5-1
Xelahes5-1.S
Xelahes5-1.L
Lachhes5sc00001
Lachhes5sc00319
Xelahes5-4.L
Xetrhes5-4
Xelahes5-4.S
Xelahes5-3.L
Xelahes5-3.S
Xetrhes5-3
Xelahes5-5.L
Xelahes5-7.L
Xetrhes5-6
Xelahes5-6.S
Xelahes5-9.L
Xelahes5-9.S
Xetrhes5-9
hes5
Xelahes5-6.L
Xelahes5-7.S
Xetrhes5-5
Xelahes5-5.S
Xetrhes5-8
Xelahes5-8.S
Xelahes5-8.L
Lachhes5sc00199
Gagahes5chr21-2
Xelahes5-2.L
Xelahes5-2.S
Xetrhes5-2
Gejahes5Sc135-2
Gagahes5chr21-1
Mumuhes5
Hosahes5
Gagahes5chr21-3
Gejahes5sc135-1
Lachhes6|102353186
Xelahes6-1.S
Xetrhes6-1
Xelahes6-1.L
Gagahes6|107051857
Mumuhes6
Hosahes6
Gejahes6|107110526
Xelahes6-2.L
Xelahes6-2.S
Xetrhes6-2
Gejahes6|107110466
Gagahes6|424829
Lachhes6|102354037
Mumuhes1
Hosahes1
Xetrhes1
Xelahes1.L
Xelahes1.S
Gejahes1
Gejahes4
Gagahes4
Hosahes4
Lachhes4
Xelahes4.L
Xetrhes4
Xelahes4.S
Gagahes1
Lachhes1|102355895
Lachhes1|102365457
Xelahes2.L
Xetrhes2
Lachhes2
Gejahes2
Hosahes2
Mumuhes2
Xelahes3.S
Xelahes3.L
Hosahes3
Mumuhes3
Xetrhes3
Xetrhes7-2
Xelahes7-2.L
Xelahes7-2.S
Gejahes7
Xelahes7-1.L
Xelahes7-1.S
Xetrhes7-1
Xelahes7-3.L
Xelahes7-3.S
Xetrhes7-3
Hosahes7
Mumuhes7

Fig.S4

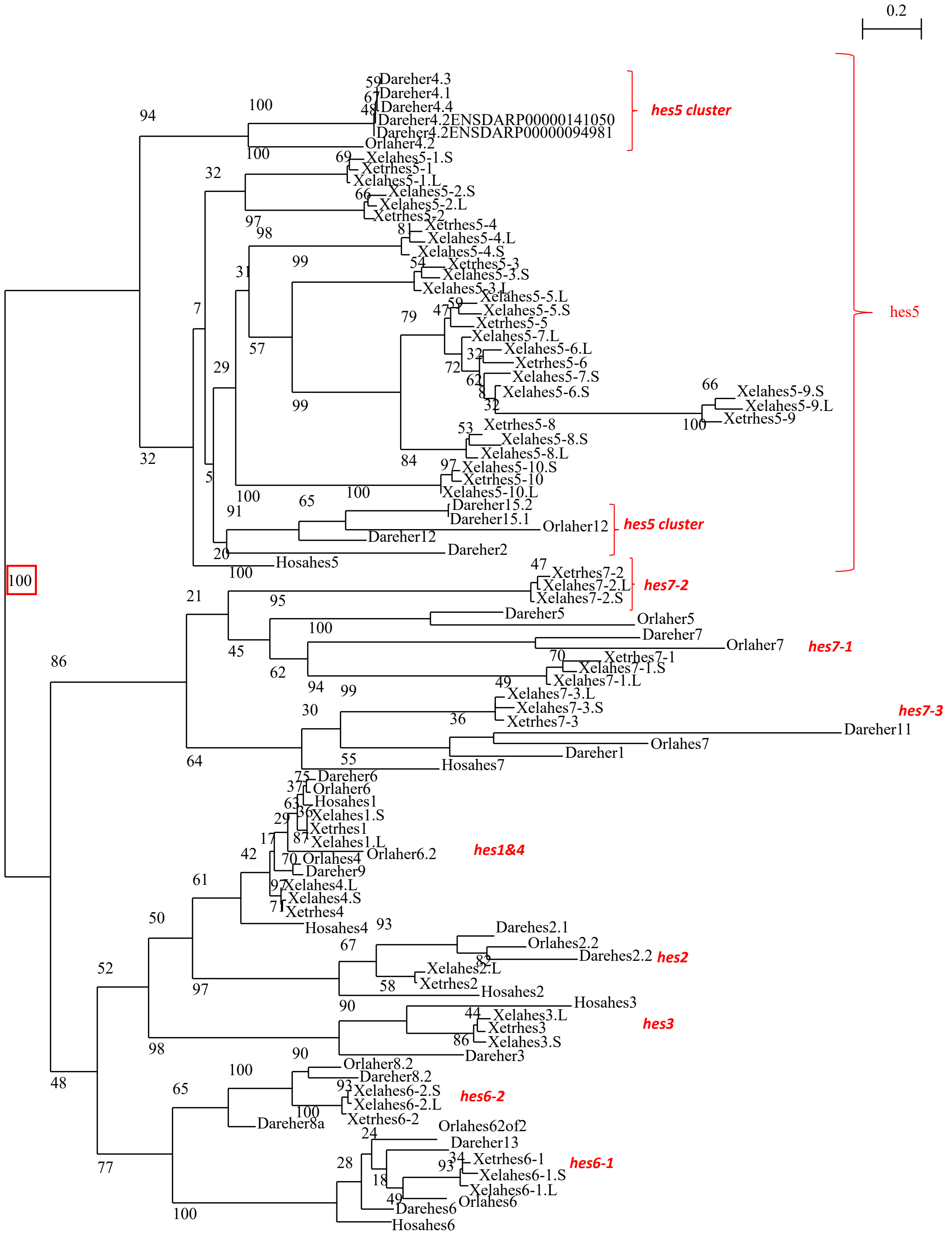
0.2
Dareher4.3
Dareher4.1
Dareher4.4
Dareher4.2ENSDARP00000141050
Dareher4.2ENSDARP00000094981
Orlaher4.2
hes5 cluster
Xelahes5-1.S
Xetrhes5-1
Xelahes5-1.L
Xelahes5-2.S
Xelahes5-2.L
Xetrhes5-2
Xetrhes5-4
Xelahes5-4.L
Xelahes5-4.S
Xetrhes5-3
Xelahes5-3.S
Xelahes5-3.L
Xelahes5-5.L
Xelahes5-5.S
Xetrhes5-5
Xelahes5-7.L
Xelahes5-6.L
Xetrhes5-6
Xelahes5-7.S
Xelahes5-6.S
Xelahes5-9.S
Xelahes5-9.L
Xetrhes5-9
Xetrhes5-8
Xelahes5-8.S
Xelahes5-8.L
Xelahes5-10.S
Xetrhes5-10
Xelahes5-10.L
Dareher15.2
Dareher15.1
Orlaher12
hes5 cluster
Dareher12
Dareher2
Hosahes5
hes5
Xetrhes7-2
Xelahes7-2.L
Xelahes7-2.S
hes7-2
Dareher5
Orlaher5
Dareher7
Orlaher7
hes7-1
Xetrhes7-1
Xelahes7-1.S
Xelahes7-1.L
Xelahes7-3.L
Xelahes7-3.S
Xetrhes7-3
hes7-3
Dareher11
Orlahes7
Dareher1
Hosahes7
Dareher6
Orlaher6
Hosahes1
Xelahes1.S
Xetrhes1
Xelahes1.L
Orlahes4
Orlaher6.2
hes1&4
Dareher9
Xelahes4.L
Xelahes4.S
Xetrhes4
Hosahes4
Darehes2.1
Orlahes2.2
Darehes2.2
hes2
Xelahes2.L
Xetrhes2
Hosahes2
Hosahes3
Xelahes3.L
Xetrhes3
hes3
Xelahes3.S
Dareher3
Orlaher8.2
Dareher8.2
Xelahes6-2.S
hes6-2
Xelahes6-2.L
Dareher8a
Xetrhes6-2
Orlahes62of2
Dareher13
Xetrhes6-1
hes6-1
Xelahes6-1.S
Xelahes6-1.L
Darehes6
Orlahes6
Hosahes6

Fig.S5

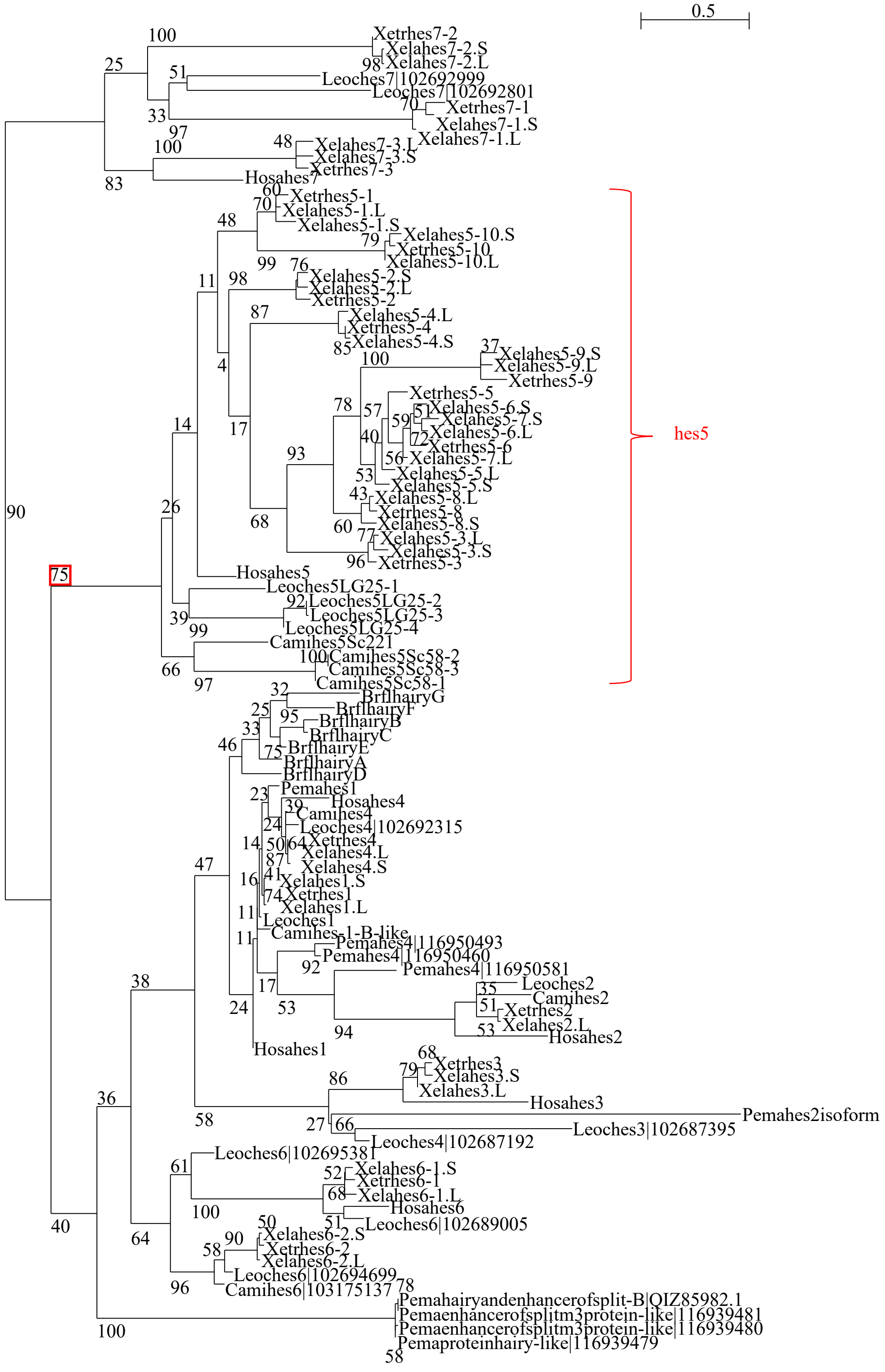
0.5
Xetrhes7-2
Xelahes7-2.S
Xelahes7-2.L
Leoches7|102692999
Leoches7|102692801
Xetrhes7-1
Xelahes7-1.S
Xelahes7-1.L
Xelahes7-3.L
Xelahes7-3.S
Xetrhes7-3
Hosahes7
Xetrhes5-1
Xelahes5-1.L
Xelahes5-1.S
Xelahes5-10.S
Xetrhes5-10
Xelahes5-10.L
Xelahes5-2.S
Xelahes5-2.L
Xetrhes5-2
Xelahes5-4.L
Xetrhes5-4
Xelahes5-4.S
Xelahes5-9.S
Xelahes5-9.L
Xetrhes5-9
Xetrhes5-5
Xelahes5-6.S
Xelahes5-7.S
Xelahes5-6.L
Xetrhes5-6
Xelahes5-7.L
Xelahes5-5.L
Xelahes5-5.S
Xelahes5-8.L
Xetrhes5-8
Xelahes5-8.S
Xelahes5-3.L
Xelahes5-3.S
Xetrhes5-3
Hosahes5
Leoches5LG25-1
Leoches5LG25-2
Leoches5LG25-3
Leoches5LG25-4
Camihes5Sc221
Camihes5Sc58-2
Camihes5Sc58-3
Camihes5Sc58-1
hes5
BrflhairyG
BrflhairyF
BrflhairyB
BrflhairyC
BrflhairyE
BrflhairyA
BrflhairyD
Pemahes1
Hosahes4
Camihes4
Leoches4|102692315
Xetrhes4
Xelahes4.L
Xelahes4.S
Xelahes1.S
Xetrhes1
Xelahes1.L
Leoches1
Camihes-1-B-like
Pemahes4|116950493
Pemahes4|116950460
Pemahes4|116950581
Leoches2
Camihes2
Xetrhes2
Xelahes2.L
Hosahes2
Hosahes1
Xetrhes3
Xelahes3.S
Xelahes3.L
Hosahes3
Pemahes2isoform
Leoches3|102687395
Leoches4|102687192
Leoches6|102695381
Xelahes6-1.S
Xetrhes6-1
Xelahes6-1.L
Hosahes6
Leoches6|102689005
Xelahes6-2.S
Xetrhes6-2
Xelahes6-2.L
Leoches6|102694699
Camihes6|103175137
Pemahairyandenhancerofsplit-B|QIZ85982.1
Pemaenhancerofsplitm3protein-like|116939481
Pemaenhancerofsplitm3protein-like|116939480
Pemaproteinhairy-like|116939479

Fig.S6

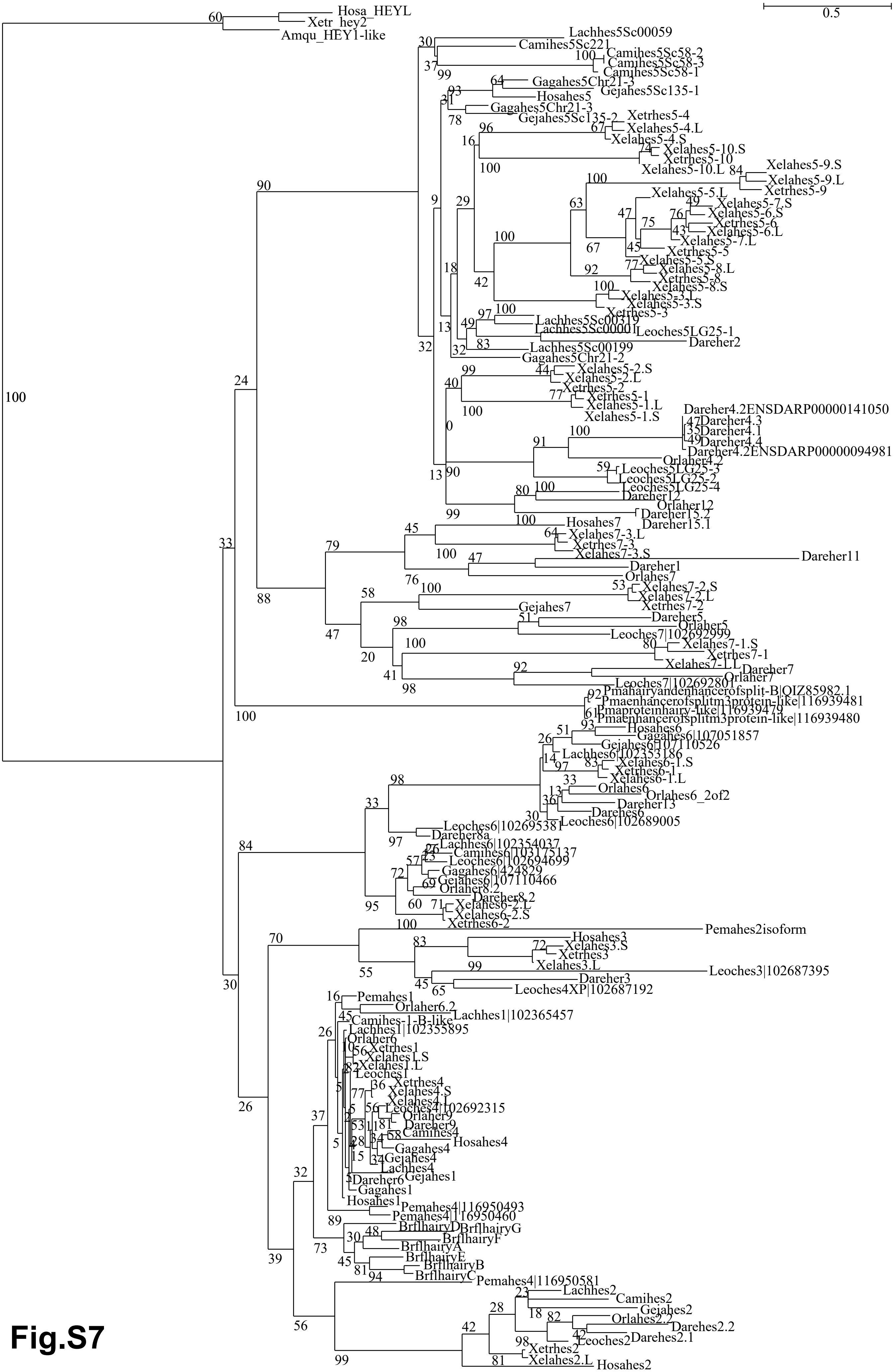

0.5
Hosa_HEYL
Xetr_hey2
Amqu_HEY1-like
Lachhes5Sc00059
Camihes5Sc221
Camihes5Sc58-2
Camihes5Sc58-3
Camihes5Sc58-1
Gagahes5Chr21-3
Gejahes5Sc135-1
Hosahes5
Gagahes5Chr21-3
Gejahes5Sc135-2
Xetrhes5-4
Xelahes5-4.L
Xelahes5-4.S
Xelahes5-10.S
Xetrhes5-10
Xelahes5-10.L
Xelahes5-9.S
Xelahes5-9.L
Xetrhes5-9
Xelahes5-5.L
Xelahes5-7.S
Xelahes5-6.S
Xetrhes5-6
Xelahes5-6.L
Xelahes5-7.L
Xetrhes5-5
Xelahes5-5.S
Xelahes5-8.L
Xetrhes5-8
Xelahes5-8.S
Xelahes5-3.L
Xelahes5-3.S
Xetrhes5-3
Lachhes5Sc00319
Lachhes5Sc00001
Leoches5LG25-1
Dareher2
Lachhes5Sc00199
Gagahes5Chr21-2
Xelahes5-2.S
Xelahes5-2.L
Xetrhes5-2
Xetrhes5-1
Xelahes5-1.L
Xelahes5-1.S
Dareher4.2ENSDARP00000141050
Dareher4.3
Dareher4.1
Dareher4.4
Dareher4.2ENSDARP00000094981
Orlaher4.2
Leoches5LG25-3
Leoches5LG25-2
Leoches5LG25-4
Dareher12
Orlaher12
Dareher15.2
Dareher15.1
Hosahes7
Xelahes7-3.L
Xetrhes7-3
Xelahes7-3.S
Dareher11
Dareher1
Orlahes7
Xelahes7-2.S
Xelahes7-2.L
Xetrhes7-2
Gejahes7
Dareher5
Orlaher5
Leoches7|102692999
Xelahes7-1.S
Xetrhes7-1
Xelahes7-1.L
Dareher7
Orlaher7
Leoches7|102692801
Pmahairyandenhancerofsplit-B|QIZ85982.1
Pmaenhancerofsplitm3protein-like|116939481
Pmaproteinhairy-like|116939479
Pmaenhancerofsplitm3protein-like|116939480
Hosahes6
Gagahes6|107051857
Gejahes6|107110526
Lachhes6|102353186
Xelahes6-1.S
Xetrhes6-1
Xelahes6-1.L
Orlahes6
Orlahes6_2of2
Dareher13
Darehes6
Leoches6|102689005
Leoches6|102695381
Dareher8a
Lachhes6|102354037
Camihes6|103175137
Leoches6|102694699
Gagahes6|424829
Gejahes6|107110466
Orlaher8.2
Dareher8.2
Xelahes6-2.L
Xelahes6-2.S
Xetrhes6-2
Pemahes2isoform
Hosahes3
Xelahes3.S
Xetrhes3
Xelahes3.L
Leoches3|102687395
Dareher3
Leoches4XP|102687192
Pemahes1
Orlaher6.2
Lachhes1|102365457
Camihes-1-B-like
Lachhes1|102355895
Orlaher6
Xetrhes1
Xelahes1.S
Xelahes1.L
Leoches1
Xetrhes4
Xelahes4.S
Xelahes4.L
Leoches4|102692315
Orlaher9
Dareher9
Camihes4
Hosahes4
Gagahes4
Gejahes4
Lachhes4
Gejahes1
Dareher6
Gagahes1
Hosahes1
Pemahes4|116950493
Pemahes4|116950460
BrflhairyD
BrflhairyG
BrflhairyF
BrflhairyA
BrflhairyE
BrflhairyB
BrflhairyC
Pemahes4|116950581
Lachhes2
Camihes2
Gejahes2
Orlahes2.2
Darehes2.2
Leoches2
Darehes2.1
Xetrhes2
Xelahes2.L
Hosahes2

Fig.S7

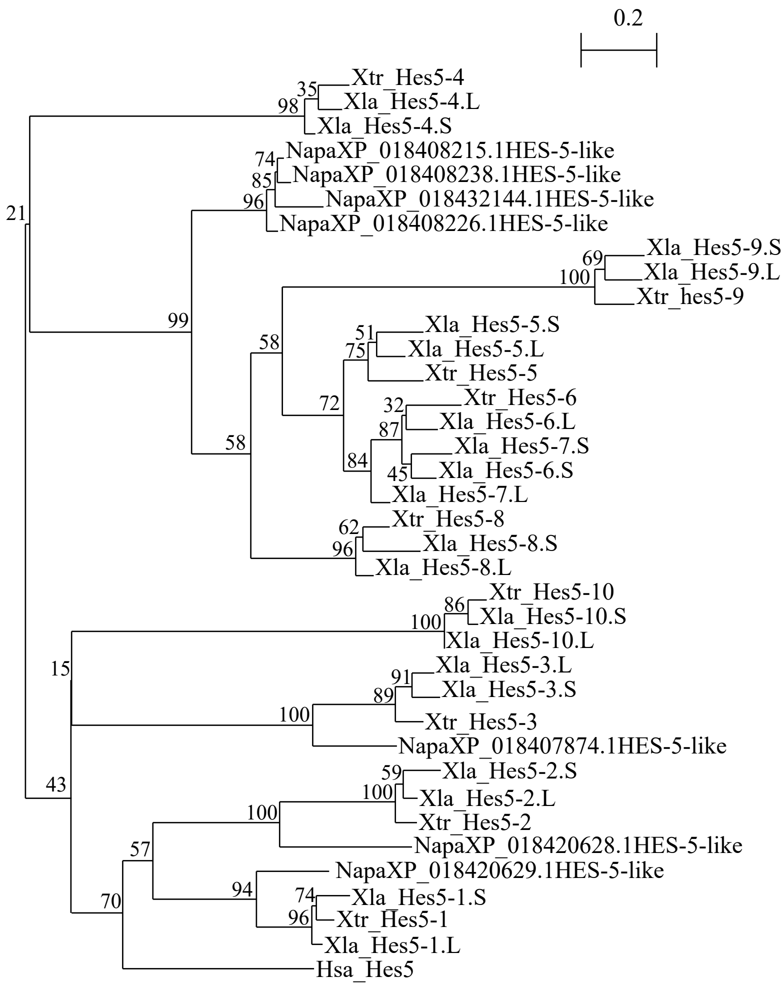
0.2
Xtr_Hes5-4
Xla_Hes5-4.L
Xla_Hes5-4.S
NapaXP_018408215.1HES-5-like
NapaXP_018408238.1HES-5-like
NapaXP_018432144.1HES-5-like
NapaXP_018408226.1HES-5-like
Xla_Hes5-9.S
Xla_Hes5-9.L
Xtr_hes5-9
Xla_Hes5-5.S
Xla_Hes5-5.L
Xtr_Hes5-5
Xtr_Hes5-6
Xla_Hes5-6.L
Xla_Hes5-7.S
Xla_Hes5-6.S
Xla_Hes5-7.L
Xtr_Hes5-8
Xla_Hes5-8.S
Xla_Hes5-8.L
Xtr_Hes5-10
Xla_Hes5-10.S
Xla_Hes5-10.L
Xla_Hes5-3.L
Xla_Hes5-3.S
Xtr_Hes5-3
NapaXP_018407874.1HES-5-like
Xla_Hes5-2.S
Xla_Hes5-2.L
Xtr_Hes5-2
NapaXP_018420628.1HES-5-like
NapaXP_018420629.1HES-5-like
Xla_Hes5-1.S
Xtr_Hes5-1
Xla_Hes5-1.L
Hsa_Hes5

Fig.S8
